# An Integrative Eco-Epidemiological Analysis of West Nile Virus Transmission

**DOI:** 10.1007/s10393-017-1249-6

**Published:** 2017-06-05

**Authors:** Annelise Tran, Grégory L’Ambert, Gilles Balança, Sophie Pradier, Vladimir Grosbois, Thomas Balenghien, Thierry Baldet, Sylvie Lecollinet, Agnès Leblond, Nicolas Gaidet-Drapier

**Affiliations:** 1 CIRAD, UPR AGIRs, Montpellier, France; 2 CIRAD, UPR TETIS, Montpellier, France; 3 EID Méditerranée, Montpellier, France; 4 Ecole Nationale Vétérinaire de Toulouse, Toulouse, France; 5 CIRAD, UMR CMAEE, Montpellier, France; 6 ANSES, Maisons-Alfort, France; 7 Université de Lyon, Marcy-l’Etoile, France; 8 INRA, Saint Genès Champanelle, France; 9 CYROI, Sainte-Clotilde, Reunion Island France

**Keywords:** disease ecology, spatial epidemiology, West Nile virus, arboviral transmission, geographic information system, modelling, Southern France, Camargue

## Abstract

West Nile disease, caused by the West Nile virus (WNV), is a mosquito-borne zoonotic disease affecting humans and horses that involves wild birds as amplifying hosts. The mechanisms of WNV transmission remain unclear in Europe where the occurrence of outbreaks has dramatically increased in recent years. We used a dataset on the competence, distribution, abundance, diversity and dispersal of wild bird hosts and mosquito vectors to test alternative hypotheses concerning the transmission of WNV in Southern France. We modelled the successive processes of introduction, amplification, dispersal and spillover of WNV to incidental hosts based on host–vector contact rates on various land cover types and over four seasons. We evaluated the relative importance of the mechanisms tested using two independent serological datasets of WNV antibodies collected in wild birds and horses. We found that the same transmission processes (seasonal virus introduction by migratory birds, *Culex modestus* mosquitoes as amplifying vectors, heterogeneity in avian host competence, absence of ‘dilution effect’) best explain the spatial variations in WNV seroprevalence in the two serological datasets. Our results provide new insights on the pathways of WNV introduction, amplification and spillover and the contribution of bird and mosquito species to WNV transmission in Southern France.

**Electronic supplementary material:**

The online version of this article (doi:10.1007/s10393-017-1249-6) contains supplementary material, which is available to authorized users.

## Introduction and Purpose

There is a growing consensus that an understanding of the interactions between hosts, vectors and pathogens is crucial to describe and predict the epidemiological dynamics of vector-borne zoonotic diseases (Allan et al. 2009; Lambin et al. 2010). To achieve such an understanding, ecology, epidemiology and geography approaches must be integrated within a cross-disciplinary research framework (Tompkins et al. 2010), as the eco-epidemiological approach proposed by Susser and Susser (1996) that takes into account multi-level factors. Such a framework is particularly relevant when dealing with complex, multi-host vector-borne diseases such as West Nile disease (WND) (Kilpatrick 2011).

WND is a mosquito-borne zoonotic disease that is caused by the West Nile virus (WNV). Wild birds are presumed to be the amplifying hosts of WNV and to contribute to its dispersal (Rappole et al. 2000; Owen et al., 2006). WNV is transmitted by ornithophilic mosquitoes between avian hosts. Virus amplification within avian and mosquito populations may lead to spillover to incidental hosts, including horses and humans. In North America, the epidemiology of WNV has received considerable attention following its emergence in 1999 and subsequent spread over the continent (Artsob et al. 2009). In Europe, however, although WNV has been reported for many years (Hubalek and Halouzka 1999), and despite a drastic increase of outbreaks since 2010 (ECDC 2015), the mechanisms of WNV transmission remain poorly understood. This is due to lack of information on the host competence (i.e. the capacity of a particular host species to infect a vector) of most Eurasian bird species, the vector competence and distribution of European mosquito species, and the environmental context promoting WNV transmission (Kilpatrick 2011).

Previous studies, most of which were conducted in North America, have proposed several mechanisms of WNV transmission involving ecological interactions between avian hosts, vectors and incidental hosts (Ezenwa et al. 2006; Kilpatrick et al. 2006; Allan et al. 2009; Loss et al. 2009). In Table 1, we review the mechanisms whereby host–vector interactions may influence various phases of WNV transmission: introduction in an area, amplification, dispersal and spillover to incidental hosts. The diversity of bird and mosquito species, their habitat preferences, seasonal fluctuations in their abundance and heterogeneity in host or vector competence may all be factors affecting host–vector transmission rates. Understanding WNV transmission thus requires bird and mosquito communities to be characterized in relation to land cover and seasonal variations.

**Table 1:** Mechanisms of WNV Transmission Between Hosts and Vectors, with Associated Predictions and Findings from this Study.

Period of the epidemiological cycle	Mechanisms	Definition and predicted patterns		Code	References	Findings from this study^a^
Introduction	Seasonal introduction by migratory birds	Migratory birds may introduce WNV in spring from endemic areas (North or sub-Saharan Africa) or in summer from epidemic areas (Eastern Europe)	Southern spring migrants	*I*_*1a*_	(Rappole et al. 2000; Owen et al., 2006; Jourdain et al. 2007; Durand et al. 2010)	++
			Eastern summer migrants	*I*_*1b*_		++
	WNV persistence in overwintering infected mosquitoes	Vectorial transmission stops during winter with mosquito’s diapause and may restart in spring when infected mosquitoes become active. WNV may persist in overwintering ornithophilic mosquitoes. Only two abundant ornithophilic mosquito species with a vector competence for WNV are found in the study area (*Culex modestus* and *Cx. pipiens*)	*Culex modestus* only	*I*_*2a*_	(Farajollahi et al. 2005; Balenghien et al. 2008)	−
			*Culex pipiens* only	*I*_*2b*_		−
			Both species	*I*_*2c*_		+
Amplification	Transmission to birds involves a single or few species of vectors	WNV amplification may involve one or both competent ornithophilic mosquito species according to differences in their feeding behaviour, competence and habitat preferences.	*Culex modestus* only	*A*_*1xx*_	(Balenghien et al. 2007; Balenghien et al. 2008)	++
			*Culex pipiens* only	*A*_*2xx*_		−
			Both species	*A*_*3xx*_		+
	Heterogeneity in avian host competence	Field and experimental studies suggest great differences between bird species in attractiveness to vectors and WNV infectiousness. In the study area, WNV have been isolated in only two bird species (House sparrows, *Passer domesticus*; and black-billed magpies, *Pica pica*).	House sparrows and black-billed magpies only	*A*_*x1x*_	(Jourdain et al. 2007)	-
			All bird species involved, heterogeneous competences among bird species	*A*_*x2x*_	(Komar et al. 2003; McKenzie and Goulet 2010)	++
			All bird species involved, homogenous competences among bird species	*A*_*x3x*_		+
	Avian host species diversity	In a heterogeneously competent bird community, high-species diversity may reduce transmission through a dilution effect because a lower proportion of mosquito would bite the most competent host species	Absence of ‘dilution effect’	*A*_*xx1*_	(Ezenwa et al. 2006; Swaddle and Calos 2008; Allan et al. 2009; Loss et al. 2009)	++
			‘Dilution effect’: diversity reduces the probability of transmission	*A*_*xx2*_		−
Dispersal	WNV-infected wild birds may disperse the virus over long distances as they move across the study area in accordance with their flying ability and propensity.				(Jourdain et al. 2007)	
Spillover	Only some vector species are likely to act as ‘bridge vectors’	One or both mosquito species may be responsible for transmitting WNV from birds to incidental hosts (horses in the study area).	*Culex modestus* only	*S*_*1*_	(Mouchet et al. 1970; Balenghien et al. 2006; Balenghien et al. 2007; Balenghien et al. 2008)	−
			*Culex pipiens* only	*S*_*2*_		−
			Both species	*S*_*3*_		+
All steps	Density-dependent transmission process	Transmission rate should increase with the local abundance of avian hosts and mosquito vectors through an increased host–vector contact rate			(McCallum et al. 2001; Shaman 2007)	

^a^The hypothesis is supported by the confrontation of one dataset (+), two datasets (++) or none (−).

To better understand the processes of WNV transmission, modelling approaches are highly complementary to experimental approaches. Several data-based studies have identified environmental features as risk factors for WNV infection, highlighting significant statistical relationships between environmental variables such as land use/land cover, climate, elevation and epidemiological data of either human cases (Cooke et al. 2006; Ruiz et al. 2007; Brown et al. 2008; Liu et al. 2009; Tran et al. 2014), equine cases (Ward 2005; Leblond et al. 2007; Mongoh et al. 2007; Pradier et al. 2008; Pradel et al. 2009; Ward et al. 2009; Chevalier et al. 2009), infected birds (Gibbs et al. 2006) or infected mosquitoes (Ezenwa et al. 2007; Ozdenerol et al. 2008). Based on those relationships, risk maps of WNV circulation are constructed; nevertheless, the underlying mechanisms driving the correlations remain often unknown (Ezenwa et al. 2007). On the other hand, several mechanistic, process-based models have been developed to address various aspects of WNV disease transmission (Lord and Day 2001; Thomas and Urena 2001; Naowarat and Tang 2004; Wonham et al., 2004; Bowman et al. 2005; Liu et al. 2006; Rappole et al. 2006; Hartemink et al. 2007; Shaman 2007; Bouden et al. 2008; Durand et al.). Yet, few of those models are spatially explicit (Liu et al. 2006; Rappole et al. 2006; Bouden et al. 2008) neither adapted to represent the host–vector contact at a local scale in a real landform (Shaman 2007).

**Figure 1:**
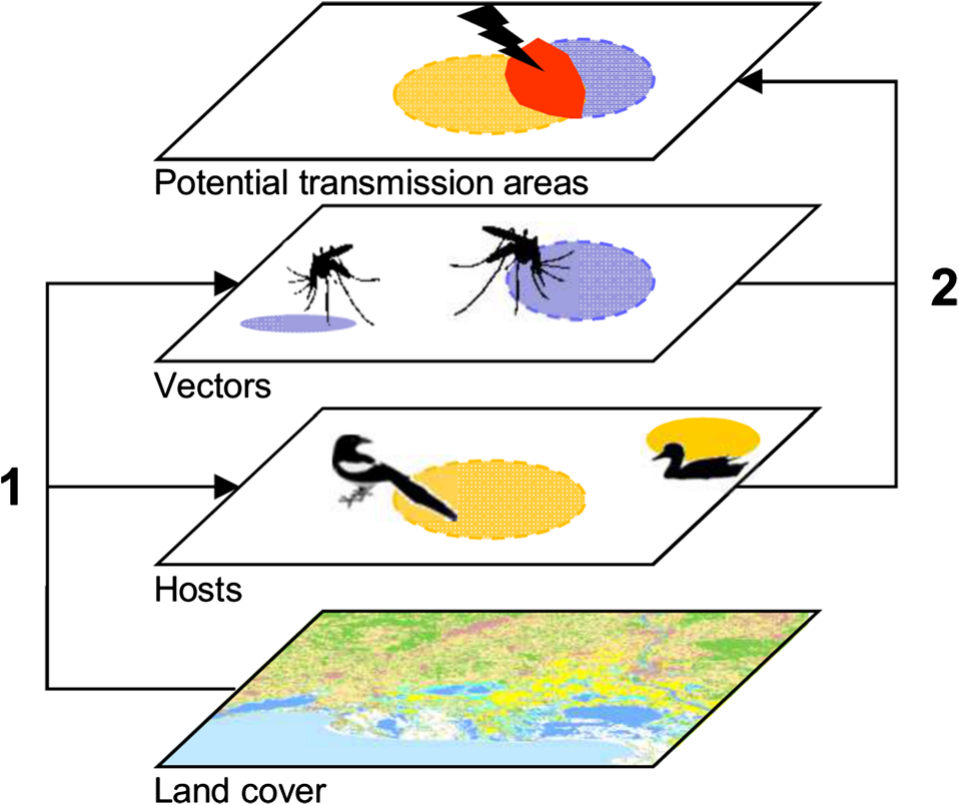
Schematic host–vector transmission process used to evaluate the distribution of West Nile virus occurrence in our analysis. (1) Land cover determines the distribution of hosts and vectors. (2) Transmission (in *red*) occurs as a result of hosts and vectors co-occurrence in space and time, their abundance and competence and host diversity (Color figure online).

In this study, we developed an original method within a spatial modelling framework to test a range of alternative hypotheses underpinning WNV introduction, amplification, dispersal and spillover in an area of Southern France where there have been recurrent outbreaks (Murgue et al. 2001; Bournez et al. 2015). Using an integrative ecological database of bird and mosquito species linked to a geographic information system (GIS), we mapped the areas where the successive steps of the WNV transmission could occur from host–vector contacts (Fig. 1) according to the different combinations of mechanisms summarized in Table 1. In this process, two synthetic indices, a WNV circulation index and a WNV spillover index, were calculated for each combination and mapped over the study area. We evaluated the relative importance of the alternative mechanisms tested by comparing through a regression model these sets of indices to independent serological datasets of WNV antibodies collected in wild birds and horses across the study area. Finally, we compared the final risk map of WNV spillover with the locations of equine outbreaks reported during the epidemics which occurred in 2015.

## Methods

### Ecological Database

**Figure 2:**
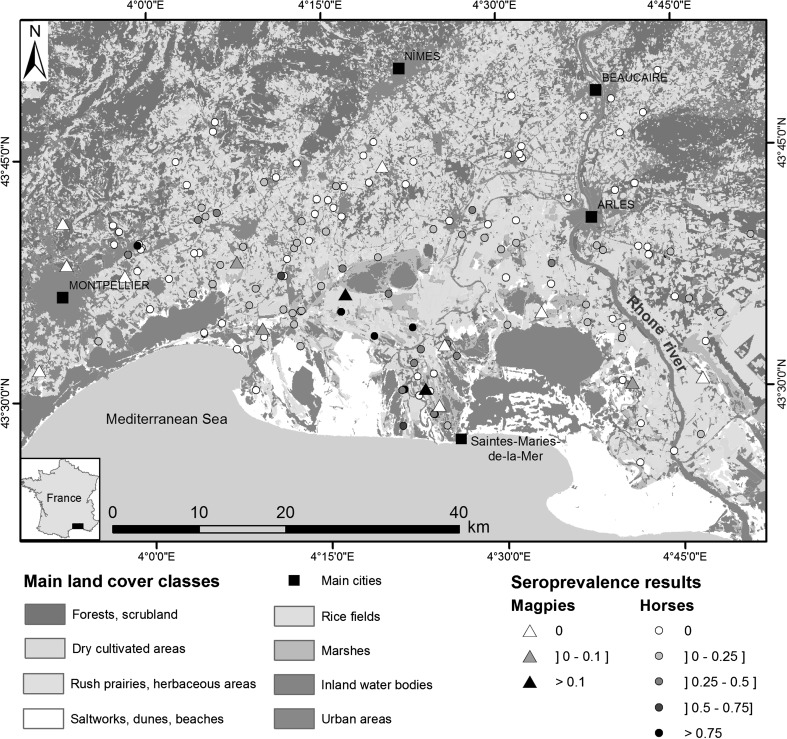
Location of the study area, Camargue region, Southern France, and results of seroprevalence studies of West Nile virus infection in magpies and horses.

The study area—the Camargue—consists in a vast river delta in Southern France (Fig. 2) characterized by a mosaic of dry (agricultural fields, scrubland, forests) and wetlands habitats (coastal lagoons, marshes, rice fields). The extent and diversity of habitats are favourable to a diversity of wild bird species (Isenmann 1993) and to the development of ornithophilic *Culex* mosquito populations (Balenghien et al. 2006).

An integrative database was built to characterize the diversity of host–vector associations over space and time. First, a list of potential mosquito vectors and avian host species was established based upon the literature. The vectors species were restricted to the two most locally abundant ornithophilic mosquito species for which a WNV competence had been evidenced (*Culex modestus* Ficalbi and *Culex pipiens* Linnaeus) (Balenghien et al. 2008). All wild bird species present in the study area, excluding rare and vagrant species, were considered as potential avian host species (180 species of 48 bird families) based on reports of the large diversity of bird species found infected with WNV (Komar et al. 2003; Jourdain et al. 2007). The host competence of each bird species was evaluated using a competence index adapted from (Komar et al. 2003) in which competence is considered to be the product of host susceptibility (exposure and receptivity to infection) and infectiousness (intensity and duration of viremia) (see Supplementary Material, Technical Appendix 1, Table S1 for details). Wild bird species were also classified according to their migratory behaviour (Cramp and Simmons 1982; Jourdain et al. 2007) in relation to areas where WNV is endemic or potentially epidemic: resident (present year-round), southern spring migrants (migratory birds arriving in spring from sub-Saharan and North African wintering quarters) and eastern summer migrants (migratory birds arriving in summer from breeding areas in North-Eastern Europe) (Supplementary Material, Technical Appendix 1, Table S2). Second, a list of common ecological units (seasons and land cover types) was defined to characterize the temporal and geographic variations in the diversity and abundance of mosquito and wild bird species over the study area. A total of 4 seasons (spring, summer, autumn and winter) and 27 land cover types were considered. Remote sensing images were used to map land cover (See Supplementary Material, Technical Appendix 2). Third, the abundance of mosquito and wild bird species was estimated for each ecological unit using an abundance index which was developed based on findings in the literature and expert opinions. Mosquito and wild bird distribution databases were linked to the land cover map for each season within a GIS (Supplementary Material, Technical Appendix 3). Finally, field bird counts and mosquito trapping exercises were conducted to validate the expert knowledge-based databases. The spatial and seasonal variations in bird and mosquito abundance measured in the field were predicted well by the abundance indices (Supplementary Material, Technical Appendix 4).

### Spatial Analysis Procedures to Predict Areas of WNV Transmission

A transmission of WNV was considered to happen when host and vector species co-occurred in the same land cover class and season (Fig. 1), with an intensity related to the relative competence, abundance and diversity of hosts and vectors sharing the same ecological unit.

#### Scenarios

We defined a ‘scenario’ of WNV transmission as a combination of different hypotheses made for the steps of introduction, amplification/dispersal and spillover of WNV, noted *I*_*x*_ *A*_*xxx*_ *S*_*x*_, according to the codes listed in Table 1 (for example, the scenario *I*_*1a*_ *A*_*111*_ *S*_*1*_ is the scenario resulting from the combination of *I*_*1a*_: WNV introduction by southern spring migratory birds, *A*_*1xx*_: WNV amplification by *Cx. modestus* as vectors, *A*_*x1x*_: house sparrows and black-billed magpies as hosts, *A*_*xx1*_: no ‘dilution effect’, and *S*_*1*_: WNV spillover by *Cx. modestus*).

**Figure 3:**
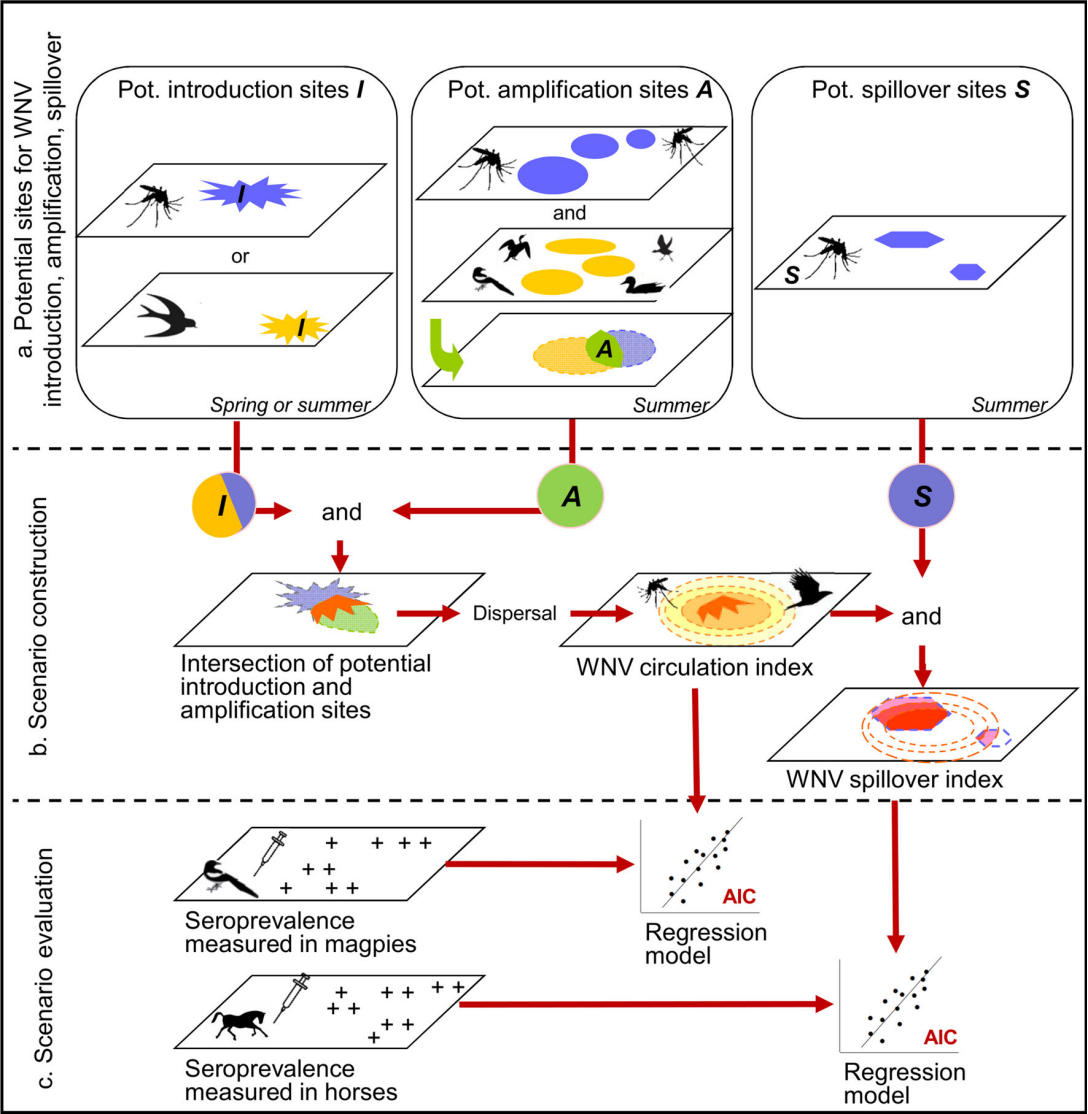
Conceptual representation of the different steps to model and evaluate different possible West Nile virus (WNV) scenarios in a geographic information system environment. (**a**) Potential sites for WNV introduction, amplification/dispersal and spillover are mapped; (**b**) maps of introduction, amplification/dispersal and spillover sites are combined to map WNV circulation and spillover indices; (**c**) different scenarios are evaluated by confrontation of WNV circulation and spillover indices with seroprevalence data measured in magpies and horses.

We used GIS spatial analysis tools (overlay intersection operators, spatial selections and distance calculations) to generate the maps derived from all of the possible scenarios following the steps detailed below (Fig. 3).

#### Mapping Potential Sites for WNV Introduction

‘Areas of potential WNV introduction’ (*I*) were defined as areas where either (1) overwintering mosquitoes occur in spring, (2) southern bird migrants occur in spring, or (3) eastern bird migrants occur in summer (Table 1; Fig. 3a). Both *Cx. modestus* and *Cx. pipiens* mosquito species were considered (alternatively or concomitantly) as competent vectors for the overwintering process. The potential for virus introduction was evaluated for each location in the study area according to the sum of the abundance indices of migratory bird species present in spring or in summer and alternatively according to the sum of abundance indices of mosquito species present in autumn before the mosquitoes’ diapause, reclassified as semiquantitative indices (null, low, moderate, high) (Supplementary Material, Technical Appendix 5.1).

#### Mapping Potential Sites for WNV Amplification/Dispersal

Amplification was considered to occur in areas where WNV vectors and hosts are present in summer (‘areas of potential amplification’ noted *A*). One or both mosquito species were considered, in association with bird species according to their relative competence and abundance (Table 1; Fig. 3a). The potential for virus amplification was evaluated as the product of an index of potential amplification by the vectors, an index of potential amplification by the hosts and an index taking into account a possible ‘dilution effect’, decreasing when the abundance of the least competent host species increases (Supplementary Material, Technical Appendix 5.2). It was reclassified as semiquantitative indices to reflect the probability (null, low, moderate, high) of WNV amplification from host–vectors contacts. The dispersal of WNV outside a location where amplification had occurred was evaluated according to the potential dispersal range of the bird and mosquito species: an active dispersal distance of 500 metres was considered for all mosquito species (Service 1997); for birds, the local dispersal range was estimated for each species using findings from the literature (Technical Appendix 1, Table S3).

#### Mapping Potential Sites for WNV Spillover

Spillover from bird to incidental hosts was considered to occur mainly in summer with either *Cx. modestus* or *Cx. pipiens* acting as bridge vector species (Table 1; Fig. 3a). The ‘potential spillover areas’ (*S*) were defined as sites where each or both vector species are present in summer. The potential for virus spillover was evaluated according to the sum of the abundance indices of the mosquito species, reclassified as semiquantitative indices (null, low, moderate, high) (Supplementary Material, Technical Appendix 5.3).

#### Calculation of a WNV Circulation Index

The predicted areas of introduction (*I*) were intersected with the areas of amplification (*A*) to map the areas of WNV introduction followed by an amplification. The resulting risk of WNV introduction amplification was obtained by the multiplication of the potentials for virus introduction and for virus amplification. A WNV circulation index was defined as the risk level of WNV circulation in wild birds resulting from each scenario after the steps of introduction and amplification/dispersal (Fig. 3b), taking into account the dispersal range associated with each species involved in the amplification. It thus decreases with the distance to the areas of virus introduction amplification (Supplementary Material, Technical Appendix 5.4).

#### Calculation of a WNV Spillover Index

The WNV circulation index map was intersected with the areas favourable to WNV spillover (*S*) to estimate a WNV spillover index (Fig. 3b).

### Evaluation of Scenario Predictions

We used two independent serological datasets of WNV antibodies collected in the study area to evaluate the predictions from the various scenarios at two different steps of the transmission process: (1) virus amplification using bird serological data and (2) virus spillover using horses serological data (Fig. 3c).

#### Wild Bird Serological Data

We used seroprevalence data on black-billed magpies (*Pica pica*) collected during serological surveys conducted between 2004 and 2007 (Jourdain et al. 2007; Balança et al. 2009). Magpies are good sentinels of WNV circulation for several reasons: this species belongs to the Corvidae, a family associated with high WNV infection rates and host competence (Komar et al. 2003; Kilpatrick et al. 2006); WNV isolation and high WNV seroprevalence have been reported in the Camargue area (Jourdain et al. 2008); the species is ubiquitous, present year-round and highly territorial. During the serological surveys, free-living magpies (*n* = 285) were captured using corvid baited traps at 15 sites over the study area (Fig. 2). Their ages were calculated from plumage characteristics (immature: first to second year, adults: ≥third year). A blood sample was collected and screened for WNV-specific immunoglobulin G using standard diagnostic procedures (Jourdain et al. 2007; Balança et al. 2009).

#### Equine Serological Data

We used data from a serological survey conducted on horses in the study area in 2007 and 2008 (Pradier et al. 2014). A total of 1161 horses living in the Camargue and originating from 135 stables distributed across the study area (Fig. 2) were sampled. The age of each horse was recorded. Sera were processed and tested for anti-WNV antibodies using an enzyme-linked immunosorbent assay (ELISA) (Pradier et al. 2014).

#### Statistical Analysis

Regression models were built to assess the association between (1) seroprevalence measured in magpies and the WNV circulation index and (2) seroprevalence measured in horses and the WNV spillover index (Fig. 3c). For each scenario, the mean values of WNV circulation and WNV spillover indices were extracted from the predictive maps within a 2-km radius of each location where seroprevalence was measured in birds and horses, respectively. This distance was chosen on the basis of known *Culex spp.* flight range and regular movements of horses (Balenghien et al. 2006; Pradier et al. 2014). We used a generalized linear model with the individual serological status as the binomial response and the age class and the WNV index as fixed effects. Goodness of fit of the models was assessed using the Pearson’s Chi-square statistic. We used the Akaike information criterion to compare models. Models were ranked according to ∆_AIC,_ the difference of AIC values of a given model and the model having the lowest AIC. Models where ∆_AIC_ ≤ 2 have substantial support, and those with ∆_AIC_ ≤ 7 are plausible (Burnham and Anderson 2004).

We calculated the normalized Akaike weights (*w*_AIC_) which can be interpreted as the relative likelihood of a model to be the best within the set of models tested in terms of the trade-off between fit to the data and parsimony. We estimated the relative importance of each mechanism by comparing the sum of Akaike weights (*Σω*_AiC_) among all models corresponding to a scenario which included this mechanism. Mechanisms with a high Σ*ω*_AiC_ were considered as the most likely to underpin variations in WNV seroprevalence.

## Results

### Prediction of WNV Circulation in Wild Bird Populations

The different combinations of vector and host introduction and amplification/dispersal mechanisms led to a total of 75 different scenarios and related maps of WNV circulation in wild birds. The spatial distribution and intensity of the predicted WNV circulation index varied greatly between scenarios. Two models received a substantial support (∆_AIC_ ≤ 2) from the magpie seroprevalence data. Both include the variable age class and WNV circulation index, positively associated with seroprevalence in magpies, and fit the data well (Table 2; Supplementary Material, Technical Appendix 6, Table S8).

**Table 2:** Summary of the Selection Statistics for the Top Regression Models Evaluating the Variation in Seroprevalence in Magpies and Horses in Relation to West Nile Virus Circulation and Spillover Indices, Resulting from the Different Scenarios of Introduction, Amplification/Dispersal and Spillover, Camargue Area, Southern France.

Model	Scenario	Component mechanisms					AIC	*w*_AIC_	Coefficients [95% CI]			
		Introduction	Amplification			Spillover			Intercept	Age class: adult	WNV circulation index	WNV spillover index
			Vector	Host	‘Dilution effect’							
Seroprevalence in magpies versus WNV circulation index^a^	I_1b_A_321_	Eastern summer migrants	*Culex modestus* and *Culex pipiens*	All bird species, heterogeneous competences	Absence of ‘dilution effect’		171.3	0.30	−15.23 [−15.47; −14.99]	2.51 [2.47; 2.55] (*p* < 10^−7^)	0.12 [0.12; 0.13] (*p* < 10^−4^)	
	I_1a_A_121_	Southern spring migrants	*Culex modestus* only	All bird species, heterogeneous competences	Absence of ‘dilution effect’		173.0	0.13	−14.05 [−14.29; −13.80]	2.48 [2.45; 2.52] (*p* < 10^−7^)	0.13 [0.129; 0.134] (*p* < 10^−3^)	
Seroprevalence in horses versus WNV spillover index^b^	I_1a_A_121_S_3_	Southern spring migrants	*Culex modestus* only	All bird species, heterogeneous competences	Absence of ‘dilution effect’	*Culex modestus* and *Culex pipiens*	725.5	0.57	−5.17 [−5.20; −5.14]	0.074 [0.073; 0.074] (*p* < 10^−6^)		0.064 [0.064; 0.065] (*p* < 10^−7^)

^a^Models are ordered from best to worst among a set of 75 candidate models. These two first models can be considered having substantial support (∆_AIC_ ≤ 2) and fit well the data (Pearson χ^2^ goodness-of-fit test = 317–342, ddl = 536, *p* = 1, H_0_: ‘the model fits the data’ cannot be rejected).

^b^Models are ordered from best to worst among a set of 93 candidate models. The first model can be unambiguously selected as the best model (Pearson χ^2^ goodness-of-fit test = 1053, ddl = 1066, *p* = 0.61, H_0_: ‘the model fits the data’ cannot be rejected).

Our results (Table 3) indicated that the introduction of the virus by migratory birds received much higher support (Σ*w*_AIC_ of the models with this hypothesis = 0.64) than the hypothesis of virus overwintering in mosquitoes (Σ*w*_AIC_ = 0.36). However, the relative role of southern spring migrants (Σ*w*_AIC_ = 0.25) respective to eastern summer migrants (Σ*w*_AIC_ = 0.39) cannot be distinguished from the data. For the process of amplification by vectors, the role of *Cx.* *modestus* alone, or of both *Culex* species together, received much higher support (Σ*w*_AIC_ = 0.37 and 0.50, respectively) than the role of *Cx. pipiens* alone. For birds, our results strongly suggested a heterogeneity in host competence (Σ*w*_AIC_ > 0.99). The alternative mechanism (magpies and sparrows as the only competent hosts) did not received support from the data, nor did the ‘dilution effect’ mechanism (Σ*w*_AIC_ = 0.19).

**Table 3:** Relative Importance of the Different Hypotheses of Introduction, Amplification/Dispersal and Spillover of West Nile Virus Explaining Variations in Magpies and Horses Seroprevalence Data, Camargue Area, Southern France, Based on Their Normalized Akaike Weights (*w*_AIC_).

Step	Hypothesis		Code	Magpies seroprevalence data Σ*w*_AIC_ (*n*)		Horses seroprevalence data Σ*w*_AIC_ (*n*)	
Introduction	**Introduction by migratory birds**	**Southern spring migrants**	***I***_***1a***_	**0.64 (30)**	**0.25 (15)**	**0.95**	**0.662 (21)**
		Eastern summer migrants	***I***_***1b***_		**0.39 (15)**		**0.288 (18)**
	Virus overwintering	*Culex modestus only*	*I*_*2a*_	0.36 (45)	<10^−3^ (15)	0.053	0.046 (27)
		*Culex pipiens only*	*I*_*2b*_		0.13 (15)		0.002 (12)
		Both species	*I*_*2c*_		0.23 (15)		0.002 (15)
Amplification	Vector amplification	***Culex modestus only***	***A***_***1xx***_	**0.37 (25)**		**0.85 (42)**	
		*Culex pipiens only*	*A*_*2xx*_	0.13 (25)		<10^−4^ (27)	
		Both species	*A*_*3xx*_	**0.50 (25)**		0.15 (24)	
	Host amplification	Magpies and sparrows *only*	*A*_*x1x*_	<10^−5^ (30)		<10^−3^ (0)	
		**All bird species, heterogeneous competences**	***A***_***x2x***_	**1 (30)**		**0.63 (69)**	
		All bird species, homogenous competences	*A*_*x3x*_	<10^−3^ (15)		0.37 (24)	
	Diversity effects	**Absence of ‘dilution effect’**	***A***_***xx1***_	**0.81 (45)**		**0.97 (60)**	
		‘Dilution effect’	*A*_*xx2*_	0.19 (30)		0.03 (33)	
Spillover	Spillover	*Culex modestus only*	*S*_*1*_	-		0.01 (31)	
		*Culex pipiens only*	*S*_*2*_	-		0.01 (31)	
		**Both species**	***S***_***3***_	**-**		**0.98 (31)**	

Bold text depicts the hypothesis with the higher support from the data.

*n* number of scenarios including the tested hypothesis.

### Prediction of WNV Spillover in the Equine Population

**Figure 4:**
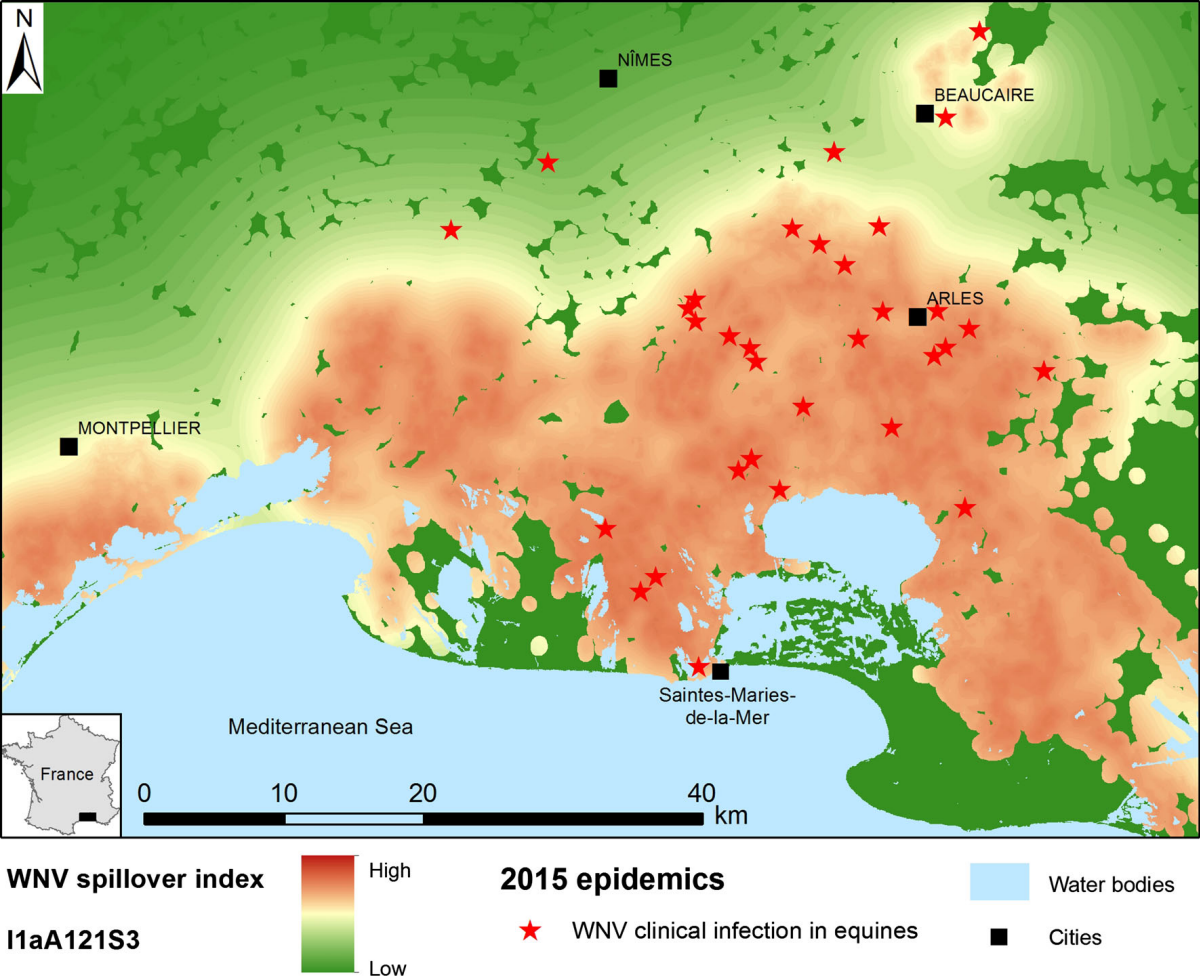
Map of areas with the highest risk of West Nile virus (WNV) spillover derived from scenario *I*_*1a*_ *A*_*121*_ *S*_*3*_ and location of clinical infection in equines, Camargue region, Southern France, 2015.

The combination of the 31 plausible (∆_AIC_ ≤ 7) maps of WNV circulation index with potential spillover areas (*S*) produced a total of 93 maps of WNV spillover index. Among this set of models, only one received substantial support (∆_AIC_ ≤ 2) from the horse seroprevalence data. This model includes the variable age class and WNV spillover index, positively associated with seroprevalence, and fits the data well (Table 2; Supplementary Material, Technical Appendix 6, Table S9). The terms of the introduction and amplification stages of the transmission process used in this model are similar to the ones from the second best bird-amplification model (Table 2). The resulting map is presented in Figure 4.

The results of the relative importance of the alternative mechanisms of WNV introduction and amplification/dispersal obtained with the horse seroprevalence data were mostly similar to those obtained with the wild bird seroprevalence data (Table 3). The highest AIC weight support was obtained for the role of migratory birds in virus introduction (Σ*w*_AIC_ = 0.95), the role of *Cx. modestus* as the main vector species for amplification (Σ*w*_AIC_ = 0.85), the heterogeneity in avian host competence (Σ*w*_AIC_ = 0.63) and the absence of any detectable ‘dilution effect’ (Σ*w*_AIC_ = 0.97). In addition, the AIC weight-based comparison procedure strongly suggested that both vector species are involved in the spillover process of WNV to horse populations (Σ*w*_AIC_ = 0.98).

Finally, we found that the map of areas with the highest risk of WNV spillover derived from the best scenario (Fig. 4) was highly consistent with the distribution of equine outbreaks reported during the last WNV epidemics in the Camargue (Bournez et al. 2015). Among the 43 clinical equine outbreaks reported in the study area, 41 (95%) were located within the areas at risk.

## Discussion

The comparison of WNV circulation and spillover indices with avian and equine seroprevalence data leads to the same conclusions about the most likely mechanisms driving virus introduction, amplification/dispersal and spillover in our study area. The results showed that while there may not be a unique scenario explaining WNV transmission, a small number of possible scenarios explain well the observed spatial heterogeneity in WNV seroprevalence. According to our analysis, some of the hypotheses tested do not fit at all with the observed seroprevalence patterns. The consistent findings from the confrontation of our spatial models as indicators of the hypothesize mechanisms of WNV transmission with independent seroprevalence and outbreak datasets strengthen our conclusions about the most likely scenario explaining the introduction, local circulation and spillover of WNV in Southern France. This modelling study is thus highly complementary to experimental approaches that are required to test the mechanisms themselves.

### Source of WNV Introduction

We found that spring and summer migratory birds, not overwintering infectious mosquitoes, are the most likely source of WNV. This result agrees with a modelling study of WNV circulation between Europe and Africa which found that overwintering mechanisms in vectors are not needed to reproduce the observed data of seroprevalence rates in migratory and resident wild birds, minimal infection rates in vectors or seroprevalence and incidence rates in horses (Durand et al. 2010). In our study, WNV introduction by southern spring bird migrants better explained seroprevalence in horses, although the role of eastern summer birds slightly better matched seroprevalence in magpies (Table 3). This suggests that either both mechanisms coexist, or that our epidemiological data were insufficient to distinguish between the two hypothesized mechanisms.

### Contribution of Different Mosquito Species to WNV Amplification and Spillover

According to our analysis, *Cx. modestus* was identified as the main amplifier of WNV in the study area compared to *Cx. pipiens*. This result corroborates the results of field investigations following recent (Balenghien et al. 2006; Leblond et al. 2007) and past WNV outbreaks in the Camargue (Hannoun et al. 1964; Mouchet et al. 1970) and of laboratory competence experiments (Balenghien et al. 2007; Balenghien et al. 2008) demonstrating that *Cx. modestus* is an extremely efficient WNV vector. However, the comparison between the WNV spillover index and seroprevalence in horses suggests that *Cx. pipiens* is, together with *Cx. modestus*, likely involved in the spillover of WNV to horses. This result could explain the previous occurrence of WND outbreaks in drier areas of the Camargue region (Durand et al. 2002) and corroborates observations of some equine or human cases diagnosed in dry areas where *Cx. modestus* are absent but large populations of *Cx. pipiens* are present. These observations suggest that *Cx. pipiens* can also be a good amplifier of WNV in other geographic contexts, such as in Italy (Romi et al. 2004), Portugal (Almeida et al. 2008) and Spain (Munoz et al. 2012).

### Contribution of Different Bird Species to WNV Amplification

The fact that sparrows and magpies are relatively abundant and widespread bird species commonly living close to human habitations may explain the detection of WNV in sick and dead birds in these two species (Jourdain et al. 2007). However, our study suggests that other bird species may play a role as indicated from both avian and equine serological data. According to our results, the hypothesis of heterogeneity in bird host competence is valid in the Camargue context. However, these results should be interpreted with caution, as the criteria used to classify the different bird species according to their host competence included experimental infection data of North American species. Indeed, we estimated avian host competence from available studies on seroprevalence and experimental infection. Only seroprevalence studies measured in Palearctic birds were considered. However, due to the paucity of European bird species that have been experimentally infected (11 species) (Hubalek et al. 2000), we also considered in our analysis results on North American species from the same bird families (25 species) (Komar et al. 2003). A difference in infectiousness between Nearctic and Palearctic birds may exist and may limit the significance of the results in our analysis.

### Host Diversity

The composition in terms of species diversity of the bird community did not seem to play a major role in the amplification of WNV in the Camargue region, unlike what was recently observed in North America (Ezenwa et al. 2006; Swaddle and Calos 2008; Allan et al. 2009; Johnson et al. 2012). This result agrees, however, with a study in the Chicago metropolitan area (Loss et al. 2009) showing no net effect of increasing species richness to WNV transmission. The use of a diversity metric taking into account bird abundance (Allan et al. 2009), instead of the species richness index, could clarify this trend.

### Predicting Risk Areas for WNV Transmission

The resulting risk map for WNV spillover fits the distribution of equine outbreaks reported during the last WNV epidemics in 2015 (Fig. 4) and thus could be used to implement risk-based surveillance of WNV in the area. This high spatial resolution map highlighting areas at risk of WNV spillover in equine and human populations at local scale complements well continental-scale risk maps derived from environmental and climatic predictors (Tran et al. 2014; Marcantonio et al. 2015). Moreover, the landscape-based approach developed in this study makes it possible to model the impacts of future land cover changes on the host–vector interactions and thus on WNV transmission. Such studies would complement previous studies examining the impact of climate change on WNV transmission (Soverow et al. 2009; Semenza et al. 2016).

### Limitations

In our study, we developed a simple and robust GIS-based method to map areas of potential WNV circulation and spillover. Different simplifying assumptions thus were made. The intensity of host–vector contact rates, vector trophic preferences and longevity, virus subtype properties as well as host defence behaviour and immunity could not be considered. Moreover, the temporal division into four seasons may be too rough to describe the high intra- and inter-annual variability of mosquito and bird dynamics. Different hypotheses of transmission were not tested here, such as bird-to-bird transmission, which can be responsible for virus overwintering (Naowarat and Tang 2004; Hartemink et al. 2007). Nevertheless, given the flexibility of GIS tools, such additional hypotheses can be readily integrated to refine the initial maps and produce corresponding risk maps according to data availability and the further development of scientific knowledge.

## Conclusions

We provided an original GIS-based framework to help understanding the complex interactions between hosts and vectors and their impact on the transmission of a multi-host pathogen, WNV. In this study, GIS modelling tools were appropriate to describe the high spatial and temporal variability of contacts between host and vector communities in Southern France and to simulate different processes likely to play a role in WNV transmission. Despite the simplifying assumptions discussed above, conclusions about the ecological mechanisms of WNV transmission could be clearly drawn from two independent seroprevalence datasets. The approach could be adapted to other European areas where WND outbreaks have recently occurred to test the mechanisms of WNV transmission and to map areas at risk of WNV transmission at the regional scale.

## Electronic supplementary material

Below is the link to the electronic supplementary material.

Supplementary material 1 (PDF 68 kb)

Supplementary material 2 (PDF 334 kb)

Supplementary material 3 (PDF 34 kb)

Supplementary material 4 (PDF 29 kb)

Supplementary material 5 (PDF 26 kb)

Supplementary material 6 (PDF 40 kb)
