# Supplementary material for: An Integrative Eco-Epidemiological Analysis of West Nile Virus Transmission
Source: Ecohealth. 2017 Jun 5;14(3):474–89. doi: 10.1007/s10393-017-1249-6 (PMC5662683; doi:10.1007/s10393-017-1249-6)
Supplement: Supplementary file 1 — Supplementary material 1 (PDF 68 kb) [file 10393_2017_1249_MOESM1_ESM.pdf]

An Integrative Eco-Epidemiological Analysis of West Nile Virus Transmission

Technical Appendix 1. West Nile virus host ecological database.

West Nile Virus (WNV) competence index of potential avian hosts in the study area

All wild bird species present in the study area, excluding rare and vagrant species, were considered as potential avian host species (180 species of 48 bird families). A host competence index was estimated for each wild bird species ranging from 1 (species likely to be of low epidemiological importance in WNV transmission) to 8 (species of very high importance in WNV transmission). Following Komar *et al.* (2003), we estimated an index of WNV host competence from the product of 1) **the susceptibility to infection** (the probability for a bird to be fed on by mosquitoes and become infected as a result of exposure to infectious vectors) and 2) **the infectiousness** (the capacity for a bird to develop and maintain an infectious viremia).

We evaluated the susceptibility to WNV infection from measures of seroprevalence estimated in wild birds in Europe and Africa from a comprehensive review of WNV infection studies. We calculated a mean seroprevalence per species as the percentage of individuals found seropositive compared with the total number of birds tested in various studies. We defined a susceptibility index with four classes based on the value of mean species seroprevalence using quantile discretization (1: ≤1%; 2:]1-5%]; 3:]5-15%]; 4: >15%) (Table S1).

We evaluated the infectiousness from measures of intensity and duration of viremia levels measured in experimental infections studies (29, 30). Since only a few European bird species have been experimentally infected, we considered in our analysis North American species from the same bird families. We calculated an index of infectiousness as the product of the 'mean infectiousness (*i*)' (i.e. the proportion of exposed vectors that become infectious per

day) and the ‘mean duration of viremia (days) (*d*)’ defined by Komar *et al.* (2003) and measured for 25 experimentally-infected bird species. We considered two classes of infectiousness (1: ≤1; 2: >1). We also used measures of viremia in European bird species reviewed by Hubalek (2000), considering an infectiousness class of ‘2’ for the species for which viremia was estimated to be sufficient to infect competent mosquito vectors (Table S1). Since seroprevalence and viremia measures were available for a limited number of species, information was synthesized at the family level. A mean susceptibility index and infectiousness index were calculated for each family. A final index of host competence was calculated as the product of the mean susceptibility index and the mean infectiousness index. For the few families (n=6) for which no data was available, we used the values from the most phylogenetically closely related bird family (Table S1).

Migratory behaviour of potential avian hosts in the study area

Wild bird species were also classified according to their migratory behaviour (Cramp and Simmons, 1982; Jourdain, Schuffenecker et al., 2007) in relation to areas where WNV is endemic or potentially epidemic: resident (present year-round), southern spring migrants (migratory birds arriving in spring from sub-Saharan and North African wintering quarters) and eastern summer migrants (migratory birds arriving in summer from breeding areas in North-Eastern Europe) (Table S2).

Local dispersal range of potential avian hosts in the study area

The local dispersal range of potential avian host species was estimated for each species using findings from the literature (Table S3).

Table S1. Summary table of West Nile Virus (WNV) host competence index estimated for each wild bird family from the product of the susceptibility index and the infectiousness index.

	Bird family	Nb. species	Pos. samples	Nb. samples	Mean preva-lence	Indices			References
						Susc.	Infect.	Competence	
1	*Anatidae*	13	25	307	8.1%	2	2	4	(1-10)
2	*Phasianidae*	2	0	5	0.0%	1	2	2	(5, 8, 11)
3	*Podicipedidae*	3	0	10	0.0%	1	1	1	(1, 6)
4	*Phalacrocoracidae*	1	7	25	28.0%	4	1	4	(1, 3, 5, 7)
5	*Ardeidae*	9	25	305	8.2%	3	2	6	(1-3, 5, 8, 12, 13)
6	*Ciconiidae*	1	163	1399	11.7%	3	2	6	(2, 3, 9, 10, 14-16)
7	*Phoenicopteridae*	1	38	401	9.5%	3	1	3	(1, 2, 9, 17)
8	*Accipitridae*	7	2	93	2.2%	2	1	2	(3, 5, 8, 10)
9	*Falconidae*	3	92	285	32.3%	4	2	8	(2, 3, 8, 14, 18)
10	*Rallidae*	3	132	308	42.9%	4	2	8	(1-3, 6, 13)
11	*Otididae*	1		0		1	1	1	(3)
12	*Haematopodidae*	1		0		1	1	1	See *Recurvirostridae*
13	*Recurvirostridae*	2	0	3	0.0%	1	1	1	(1, 2)
14	*Burhinidae*	1	1	4	25.0%	4	1	4	(2)
15	*Charadriidae*	6	0	9	0.0%	1	2	2	(1, 2, 13)
16	*Scolopacidae*	18	10	285	3.5%	2	2	4	(1-3, 6, 13, 19)
17	*Laridae*	12	43	602	7.1%	3	2	6	(1-3, 5-7, 9, 14, 15, 17, 20)
18	*Columbidae*	4	104	372	28.0%	4	2	8	(2-5, 7, 8, 11, 15, 20, 21)
19	*Cuculidae*	2	0	5	0.0%	1	1	1	(13)
20	*Strigidae*	5	2	34	5.9%	3	1	3	(3, 5, 13, 14, 21)
21	*Caprimulgidae*	1	1	3	33.3%	4	1	4	(2, 13)
22	*Apodidae*	1	0	14	0.0%	1	1	1	(2)
23	*Alcedinidae*	1	0	5	0.0%	1	1	1	(6, 22)
24	*Meropidae*	1	0	4	0.0%	1	1	1	(13)
25	*Coracidae*	1		0		1	1	1	See *Meropidae*
26	*Upupidae*	1	1	10	10.0%	3	1	3	(8, 11, 12)
27	*Picidae*	3	1	18	5.6%	3	1	3	(2, 3, 21)
28	*Alaudidae*	3	0	1	0.0%	1	1	1	(2)
29	*Hirundinidae*	3	5	1787	0.3%	1	2	2	(2, 3, 6, 13, 14, 19, 23)
30	*Motacillidae*	5	3	147	2.0%	2	2	4	(2, 10, 13, 19)
31	*Troglodytidae*	1		0		1	1	1	See *Prunellidae*
32	*Prunellidae*	1	0	1	0.0%	1	1	1	(13)
33	*Turdidae*	13	23	647	3.6%	2	2	4	(2, 3, 5, 6, 10-13, 19, 21, 22, 24)
34	*Sylviidae*	20	45	1801	2.5%	2	2	4	(2, 3, 6, 10-14, 19, 20, 22)
35	*Muscicapidae*	2	4	398	1.0%	2	1	2	(2, 3, 10-12)
36	*Regulidae*	2		0		2	1	2	See *Sylviidae*
37	*Timalidae*	1	0	1	0.0%	1	1	1	(6)
38	*Aegithalidae*	1		0		3	1	3	See *Paridae*
39	*Paridae*	2	2	15	13.3%	3	1	3	(2, 6, 13, 14)
40	*Remizidae*	1	1	14	7.1%	3	1	3	(6)
41	*Certhidae*	1		0		3	1	3	See *Paridae*
42	*Oriolidae*	1	0	1	0.0%	1	1	1	(11)
43	*Laniidae*	3	5	54	9.3%	3	1	3	(6, 10-13, 20)
44	*Corvidae*	4	232	1936	12.0%	3	2	6	(2-5, 8, 14, 15, 20, 21, 25)
45	*Sturnidae*	1	7	214	3.3%	2	2	4	(3, 5, 6, 13, 20, 21)
46	*Passeridae*	2	38	2581	1.5%	2	2	4	(2, 5, 8, 11, 13, 14, 20, 22, 23)
47	*Fringillidae*	5	0	167	0.0%	1	2	2	(2, 3, 11, 13, 14, 22)
48	*Emberizidae*	4	1	36	2.8%	2	1	2	(6, 20)

Table S2. Summary table of wild birds' migratory behaviour, Camargue region, Southern France.

	Number of species
Southern spring migrants only	28
Eastern summer migrants only	34
Both southern spring and eastern summer migrants	86
Resident	32

Table S3. Summary table of wild birds'potential local dispersal range during a given season, Camargue region, Southern France.

	Number of species			
Spread distance range*	Spring	Summer	Autumn	Winter
< 0.5 km	55	0	0	14
< 1 km	18	7	5	13
< 10 km	26	26	11	36
< 50 km	5	30	8	51
≥ 50 km	68	103	124	0

***** the dispersal range was estimated for all bird species from the literature (26-28) and expert knowledge.
