# Supplementary material for: An Integrative Eco-Epidemiological Analysis of West Nile Virus Transmission
Source: Ecohealth. 2017 Jun 5;14(3):474–89. doi: 10.1007/s10393-017-1249-6 (PMC5662683; doi:10.1007/s10393-017-1249-6)
Supplement: Supplementary file 2 — Supplementary material 2 (PDF 334 kb) [file 10393_2017_1249_MOESM2_ESM.pdf]

An Integrative Eco-Epidemiological Analysis of West Nile Virus Transmission

Technical Appendix 2. Land cover types and seasons used to characterize the temporal and geographical variations in diversity and abundance of mosquito and wild bird species

Seasons were defined in accordance with the timing of the life-cycle of both birds (migration, reproduction) and mosquitoes (diapause, reproduction and feeding behavior).

A total of 27 land cover types were considered that belonged to five main ecotypes: maritime areas (sea, beaches and herbaceous dunes, dune forest, lagoons, salty bare soil, salt marsh vegetation, salt works), natural coastal areas (clear water, floating aquatic vegetation, mud flat, reed beds, marshes with scirpus and rushes, rush wetland temporarily flooded, rush prairies never flooded), natural continental areas (forest, scrubland, riparian forest), agricultural areas (dry herbaceous areas, vineyard, fruit trees, rice fields, cereals, fallow land, bare soil, hedges) and built-up areas (dense urban areas, suburban and rural areas). Two Landsat Enhanced Thematic Mapper (ETM+) images (21 July and 25 October 2001) were processed to map the different land cover types within the study area, with a spatial resolution of 30 meters, through a supervised object-oriented classification process (Figure S1) (Tran et al., 2007).

References

Tran A, Gaidet N, L'Ambert G, Balenghien T, Balança G, Chevalier V, Soti V, Ivanes C, Etter E, Schaffner F, Baldet T, De la Rocque S (2007) The use of remote sensing for the ecological description of multi-host disease systems: a case study on West Nile virus in southern France. Veterinaria Italiana. 43(3): 687-697.

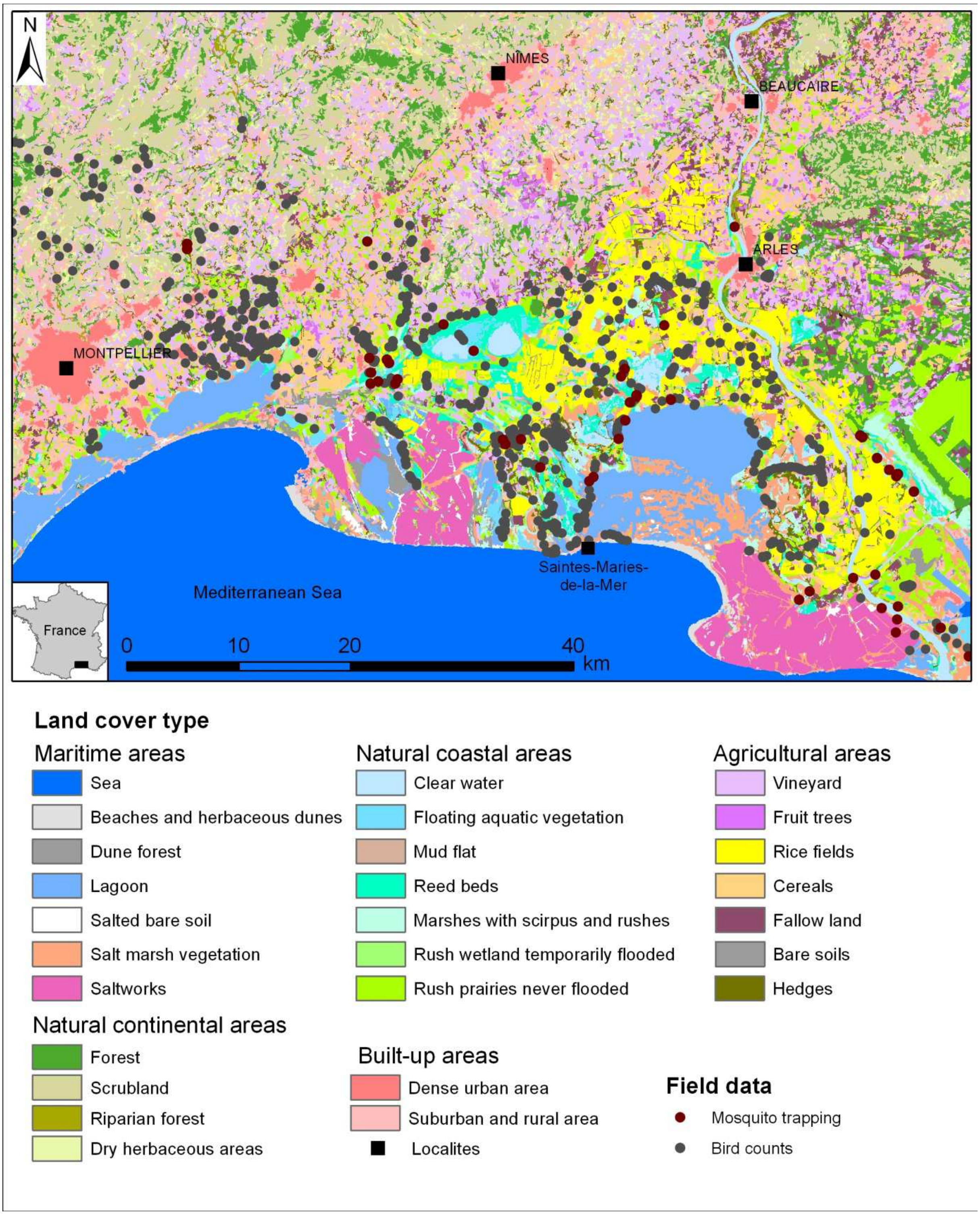

Figure S1. Land cover map of the study area, Camargue region, Southern France.
