# Supplementary material for: An Integrative Eco-Epidemiological Analysis of West Nile Virus Transmission
Source: Ecohealth. 2017 Jun 5;14(3):474–89. doi: 10.1007/s10393-017-1249-6 (PMC5662683; doi:10.1007/s10393-017-1249-6)
Supplement: Supplementary file 3 — Supplementary material 3 (PDF 34 kb) [file 10393_2017_1249_MOESM3_ESM.pdf]

An Integrative Eco-Epidemiological Analysis of West Nile Virus Transmission

Technical Appendix 3. Expert-based distribution and abundance of *Culex* mosquitoes and wild birds

3.1 *Culex* mosquitoes

The abundance of the two vectors species, *Culex modestus* and *Cx. pipiens*, was evaluated according to five index classes (0: absent; 1: very low densities; 2: low densities; 3: high densities; 4: very high densities) in each land cover type and for each season, based on literature and expert opinions (Rioux and Arnold, 1955; Rioux, 1958; Service, 1969; Mouchet et al., 1970; Savage et al., 1999; Vinogradova, 2000; Medlock et al., 2005; Balenghien et al., 2006). Considering the number of host-seeking mosquito females in a bird-baited trap per collection night, the five classes were defined as follows: absent: never collected; very low densities: few individuals collected; low densities: 10 to 40 individuals collected; high densities: 40 to 100 individuals collected; very high densities: >100 individuals collected.

For each land cover type, we considered two distinct activities to map: *i)* ‘breeding areas’, defined as areas where water bodies of different types, such as rice fields, water ponds, marshes, etc, are available for mosquito females to lay their eggs, and *ii)* ‘host-seeking areas’, defined as areas where mosquito females do not oviposit but where they can move to seek a host to take the blood meal necessary to develop their eggs. “Urban areas” (dense urban areas and suburban areas) were identified as potential breeding areas for *Cx. pipiens*, as this species may breed in small containers likely to be present in these classes (Table S4).

We then used the mosquito database information and GIS spatial analysis tools (spatial selections) to map the most realistic distribution of mosquitoes for each season and each species, taking into account the dispersal distance around the breeding areas. This active dispersal for mosquitoes usually consists of relatively short distances (about a few hundred

meters), although longer flights of >1 km may occur if hosts and oviposition sites are widely separated (Service, 1997). Here we considered that mosquitoes can disperse from the breeding areas into favourable host-seeking areas neighbouring the breeding areas.

For each species and season, the land cover map (Technical Appendix 2, Figure S1) was thus reclassified to produce a mosquito abundance index map:

- each land cover object was classified as 'breeding site', 'host-seeking site' or 'other' according to its land cover type and the mosquito database,
- a mosquito abundance index value was attributed to each land cover object: 'breeding site' objects and 'host-seeking site' objects neighbouring 'breeding sites', according to the land cover type and the associated mosquito data; the null-value was attributed to all other objects.

3.2. Wild birds

The abundance of each of the 180 wild bird species (*i.e.* all wild bird species present in the study area excluding rare and vagrant species) was evaluated according to four index classes (0: absent - the species is never or accidentally observed; 1: uncommon; 2: frequent; 3: common) in each land cover type and for each season based on ornithologist expert opinions.

References

Balenghien T, Fouque F, Sabatier P, Bicout DJ (2006) Horse-, bird-, and human-seeking behavior and seasonal abundance of mosquitoes in a West Nile Virus focus of southern France. Journal of Medical Entomology. 43: 936-946.

Medlock JM, Snow KR, Leach S (2005) Potential transmission of West Nile virus in the British Isles: an ecological review of candidate mosquito bridge vectors. Med Vet Entomol. 19(1): 2-21.

Mouchet J, Rageau J, Laumond C, Hannoun C, Beytout D, Oudar J, Corniou B, Chippaux A (1970) Epidémiologie du virus West Nile: étude d'un foyer en Camargue V. Le vecteur: Culex modestus Ficalbi Diptera; Culicidae. Annales de l'Institut Pasteur (Paris). 118: 839-855.

Rioux JA (1958) Les Culicidés du "Midi méditerranéen". Etude systématique et écologique. Paris, France, Editions Paul Lechevalier.

Rioux JA, Arnold M (1955) Les Culicidés de Camargue (étude systématique et écologique). Paris, France, Société Nationale d'Acclimatation

Savage HM, Ceianu C, Nicolescu G, Kearabatsos N, Lanciotti R, Vladimirescu A, Laiv L, Ungureanu A, Romanca C, Tsai TF (1999) Entomological and avian investigation of an epidemic of West Nile fever in Romania in 1996, with serologic and molecular characterization of a virus isolate from mosquitoes. American Journal of Tropical Medicine and Hygiene. 61: 600-611.

Service MW (1969) Observations on the ecology of some British mosquitoes. Bulletin of Entomological Research. 59: 161–194.

Service MW (1997) Mosquito (Diptera: Culicidae) dispersal--the long and short of it. J Med Entomol. 34(6): 579-588.

Vinogradova EB (2000) Culex pipiens pipiens mosquitoes: taxonomy, distribution, ecology, physiology, genetics, applied importance and control. SoÞa-Moscow, Russia, PENSOFT Publishers.

Table S4. *Culex modestus* and *Culex pipiens* breeding sites, Camargue region, Southern France.

Species	Season	Habitat	Activity	Occurence
Culex modestus	Spring	Sea	Breeding	absent
Culex modestus	Spring	Beaches and herbaceous dunes	Breeding	absent
Culex modestus	Spring	Dune forest	Breeding	absent
Culex modestus	Spring	Salt water lagoon	Breeding	uncommon
Culex modestus	Spring	Salty bare soil	Breeding	absent
Culex modestus	Spring	Salt marsh vegetation	Breeding	uncommon
Culex modestus	Spring	Floating aquatic vegetation	Breeding	uncommon
Culex modestus	Spring	Open water (fresh water)	Breeding	uncommon
Culex modestus	Spring	Mud flats with low salinity	Breeding	absent
Culex modestus	Spring	Reed beds	Breeding	frequent
Culex modestus	Spring	Marshes with scirpus and rushes	Breeding	uncommon
Culex modestus	Spring	Rush wetland temporarily flooded	Breeding	absent
Culex modestus	Spring	Rush prairies never flooded	Breeding	absent
Culex modestus	Spring	Dry herbaceous areas	Breeding	absent
Culex modestus	Spring	Riparian forest	Breeding	uncommon
Culex modestus	Spring	Hedges	Breeding	absent
Culex modestus	Spring	Forest	Breeding	absent
Culex modestus	Spring	Scrubland	Breeding	absent
Culex modestus	Spring	Vineyard	Breeding	absent
Culex modestus	Spring	Bare soil	Breeding	absent
Culex modestus	Spring	Fruit trees	Breeding	absent
Culex modestus	Spring	Rice fields	Breeding	frequent
Culex modestus	Spring	Cereals	Breeding	absent
Culex modestus	Spring	Fallow land	Breeding	absent
Culex modestus	Spring	Dense urban areas	Breeding	absent
Culex modestus	Spring	Suburban areas	Breeding	absent
Culex modestus	Spring	Saltworks	Breeding	absent
Culex modestus	Summer	Sea	Breeding	absent
Culex modestus	Summer	Beaches and herbaceous dunes	Breeding	absent
Culex modestus	Summer	Dune forest	Breeding	absent
Culex modestus	Summer	Salt water lagoon	Breeding	uncommon
Culex modestus	Summer	Salty bare soil	Breeding	absent
Culex modestus	Summer	Salt marsh vegetation	Breeding	frequent
Culex modestus	Summer	Floating aquatic vegetation	Breeding	frequent
Culex modestus	Summer	Open water (fresh water)	Breeding	frequent
Culex modestus	Summer	Mud flats with low salinity	Breeding	uncommon
Culex modestus	Summer	Reed beds	Breeding	abundant
Culex modestus	Summer	Marshes with scirpus and rushes	Breeding	frequent
Culex modestus	Summer	Rush wetland temporarily flooded	Breeding	uncommon
Culex modestus	Summer	Rush prairies never flooded	Breeding	absent

Culex modestus	Summer	Dry herbaceous areas	Breeding	absent
Culex modestus	Summer	Riparian forest	Breeding	uncommon
Culex modestus	Summer	Hedges	Breeding	absent
Culex modestus	Summer	Forest	Breeding	uncommon
Culex modestus	Summer	Scrubland	Breeding	absent
Culex modestus	Summer	Vineyard	Breeding	absent
Culex modestus	Summer	Bare soil	Breeding	absent
Culex modestus	Summer	Fruit trees	Breeding	absent
Culex modestus	Summer	Rice fields	Breeding	common
Culex modestus	Summer	Cereals	Breeding	absent
Culex modestus	Summer	Fallow land	Breeding	absent
Culex modestus	Summer	Dense urban areas	Breeding	uncommon
Culex modestus	Summer	Suburban areas	Breeding	uncommon
Culex modestus	Summer	Saltworks	Breeding	absent
Culex modestus	Autumn	Sea	Breeding	absent
Culex modestus	Autumn	Beaches and herbaceous dunes	Breeding	absent
Culex modestus	Autumn	Dune forest	Breeding	absent
Culex modestus	Autumn	Salt water lagoon	Breeding	absent
Culex modestus	Autumn	Salty bare soil	Breeding	absent
Culex modestus	Autumn	Salt marsh vegetation	Breeding	uncommon
Culex modestus	Autumn	Floating aquatic vegetation	Breeding	uncommon
Culex modestus	Autumn	Open water (fresh water)	Breeding	uncommon
Culex modestus	Autumn	Mud flats with low salinity	Breeding	absent
Culex modestus	Autumn	Reed beds	Breeding	uncommon
Culex modestus	Autumn	Marshes with scirpus and rushes	Breeding	uncommon
Culex modestus	Autumn	Rush wetland temporarily flooded	Breeding	absent
Culex modestus	Autumn	Rush prairies never flooded	Breeding	absent
Culex modestus	Autumn	Dry herbaceous areas	Breeding	absent
Culex modestus	Autumn	Riparian forest	Breeding	absent
Culex modestus	Autumn	Hedges	Breeding	absent
Culex modestus	Autumn	Forest	Breeding	absent
Culex modestus	Autumn	Scrubland	Breeding	absent
Culex modestus	Autumn	Vineyard	Breeding	absent
Culex modestus	Autumn	Bare soil	Breeding	absent
Culex modestus	Autumn	Fruit trees	Breeding	absent
Culex modestus	Autumn	Rice fields	Breeding	frequent
Culex modestus	Autumn	Cereals	Breeding	absent
Culex modestus	Autumn	Fallow land	Breeding	absent
Culex modestus	Autumn	Dense urban areas	Breeding	absent
Culex modestus	Autumn	Suburban areas	Breeding	absent
Culex modestus	Autumn	Saltworks	Breeding	absent
Culex pipiens	Spring	Sea	Breeding	absent
Culex pipiens	Spring	Beaches and herbaceous dunes	Breeding	absent
Culex pipiens	Spring	Dune forest	Breeding	absent
Culex pipiens	Spring	Salt water lagoon	Breeding	absent

Culex pipiens	Spring	Salty bare soil	Breeding	absent
Culex pipiens	Spring	Salt marsh vegetation	Breeding	absent
Culex pipiens	Spring	Floating aquatic vegetation	Breeding	uncommon
Culex pipiens	Spring	Open water (fresh water)	Breeding	frequent
Culex pipiens	Spring	Mud flats with low salinity	Breeding	uncommon
Culex pipiens	Spring	Reed beds	Breeding	uncommon
Culex pipiens	Spring	Marshes with scirpus and rushes	Breeding	uncommon
Culex pipiens	Spring	Rush wetland temporarily flooded	Breeding	uncommon
Culex pipiens	Spring	Rush prairies never flooded	Breeding	uncommon
Culex pipiens	Spring	Dry herbaceous areas	Breeding	absent
Culex pipiens	Spring	Riparian forest	Breeding	uncommon
Culex pipiens	Spring	Hedges	Breeding	absent
Culex pipiens	Spring	Forest	Breeding	uncommon
Culex pipiens	Spring	Scrubland	Breeding	absent
Culex pipiens	Spring	Vineyard	Breeding	absent
Culex pipiens	Spring	Bare soil	Breeding	absent
Culex pipiens	Spring	Fruit trees	Breeding	uncommon
Culex pipiens	Spring	Rice fields	Breeding	common
Culex pipiens	Spring	Cereals	Breeding	absent
Culex pipiens	Spring	Fallow land	Breeding	absent
Culex pipiens	Spring	Dense urban areas	Breeding	uncommon
Culex pipiens	Spring	Suburban areas	Breeding	frequent
Culex pipiens	Spring	Saltworks	Breeding	absent
Culex pipiens	Summer	Sea	Breeding	absent
Culex pipiens	Summer	Beaches and herbaceous dunes	Breeding	absent
Culex pipiens	Summer	Dune forest	Breeding	absent
Culex pipiens	Summer	Salt water lagoon	Breeding	absent
Culex pipiens	Summer	Salty bare soil	Breeding	absent
Culex pipiens	Summer	Salt marsh vegetation	Breeding	absent
Culex pipiens	Summer	Floating aquatic vegetation	Breeding	common
Culex pipiens	Summer	Open water (fresh water)	Breeding	common
Culex pipiens	Summer	Mud flats with low salinity	Breeding	uncommon
Culex pipiens	Summer	Reed beds	Breeding	common
Culex pipiens	Summer	Marshes with scirpus and rushes	Breeding	frequent
Culex pipiens	Summer	Rush wetland temporarily flooded	Breeding	frequent
Culex pipiens	Summer	Rush prairies never flooded	Breeding	uncommon
Culex pipiens	Summer	Dry herbaceous areas	Breeding	absent
Culex pipiens	Summer	Riparian forest	Breeding	common
Culex pipiens	Summer	Hedges	Breeding	absent
Culex pipiens	Summer	Forest	Breeding	frequent
Culex pipiens	Summer	Scrubland	Breeding	absent
Culex pipiens	Summer	Vineyard	Breeding	absent
Culex pipiens	Summer	Bare soil	Breeding	absent
Culex pipiens	Summer	Fruit trees	Breeding	uncommon
Culex pipiens	Summer	Rice fields	Breeding	abundant

Culex pipiens	Summer	Cereals	Breeding	absent
Culex pipiens	Summer	Fallow land	Breeding	absent
Culex pipiens	Summer	Dense urban areas	Breeding	common
Culex pipiens	Summer	Suburban areas	Breeding	abundant
Culex pipiens	Summer	Saltworks	Breeding	absent
Culex pipiens	Autumn	Sea	Breeding	absent
Culex pipiens	Autumn	Beaches and herbaceous dunes	Breeding	absent
Culex pipiens	Autumn	Dune forest	Breeding	absent
Culex pipiens	Autumn	Salt water lagoon	Breeding	absent
Culex pipiens	Autumn	Salty bare soil	Breeding	absent
Culex pipiens	Autumn	Salt marsh vegetation	Breeding	absent
Culex pipiens	Autumn	Floating aquatic vegetation	Breeding	absent
Culex pipiens	Autumn	Open water (fresh water)	Breeding	frequent
Culex pipiens	Autumn	Mud flats with low salinity	Breeding	absent
Culex pipiens	Autumn	Reed beds	Breeding	absent
Culex pipiens	Autumn	Marshes with scirpus and rushes	Breeding	uncommon
Culex pipiens	Autumn	Rush wetland temporarily flooded	Breeding	uncommon
Culex pipiens	Autumn	Rush prairies never flooded	Breeding	uncommon
Culex pipiens	Autumn	Dry herbaceous areas	Breeding	absent
Culex pipiens	Autumn	Riparian forest	Breeding	frequent
Culex pipiens	Autumn	Hedges	Breeding	absent
Culex pipiens	Autumn	Forest	Breeding	uncommon
Culex pipiens	Autumn	Scrubland	Breeding	absent
Culex pipiens	Autumn	Vineyard	Breeding	absent
Culex pipiens	Autumn	Bare soil	Breeding	absent
Culex pipiens	Autumn	Fruit trees	Breeding	absent
Culex pipiens	Autumn	Rice fields	Breeding	absent
Culex pipiens	Autumn	Cereals	Breeding	absent
Culex pipiens	Autumn	Fallow land	Breeding	absent
Culex pipiens	Autumn	Dense urban areas	Breeding	frequent
Culex pipiens	Autumn	Suburban areas	Breeding	frequent
Culex pipiens	Autumn	Saltworks	Breeding	absent
