# Supplementary material for: An Integrative Eco-Epidemiological Analysis of West Nile Virus Transmission
Source: Ecohealth. 2017 Jun 5;14(3):474–89. doi: 10.1007/s10393-017-1249-6 (PMC5662683; doi:10.1007/s10393-017-1249-6)
Supplement: Supplementary file 4 — Supplementary material 4 (PDF 29 kb) [file 10393_2017_1249_MOESM4_ESM.pdf]

An Integrative Eco-Epidemiological Analysis of West Nile Virus Transmission

Technical Appendix 4. Field assessment of the distribution and abundance of mosquitoes and birds

4.1 *Culex* mosquitoes

Mosquito captures were conducted between 2005 and 2007 using miniature CDC (Center for Disease Control) traps (John W. Hock Co., Gainesville, FL, U.S.A.) (Sudia and Chamberlain, 1962) baited with CO_2 dry ice but without light in locations protected from wind exposure (Poncon et al., 2007, L'Ambert, unpublished data). Captures were conducted overnight (from 18:00 to 10:00) for two consecutive nights with good meteorological conditions (neither rain nor wind) in 100 different sites representing eight distinct land cover types chosen to cover a range of the abundance index classes of *Culex pipiens* and *Cx. modestus* estimated in the database. These land cover types were sampled in different locations (Technical Appendix 1, Figure S1) and seasons, resulting in 451 trap-nights. Mosquito species were identified using standard morphological identification keys (Schaffner et al., 2001).

To take into account the distance at which CO_2-baited traps were most attractive to mosquitoes, an abundance index was defined as the average of the abundance class values encountered within a buffer zone around the trap location (radius=500m). The correlations between the abundance index and the observed number of mosquitoes were significant, with a better fit between the abundance index and the observed abundances for *Cx. modestus* (Spearman r=0.68, $p<10^{-5}$) than *Cx. pipiens* (Spearman r=0.58, $p<10^{-5}$).

4.2. Wild birds

A standard 15 minutes point-count method was used to assess bird species abundance (Bibby et al., 2000). Birds were identified by visual and vocal criteria. Point-count locations were selected ensuring that detection of birds was high (*e.g.* forest songbirds were only counted in

spring early in the morning when songs are the most frequent), and were located in order to cover most of the study area (Technical Appendix 1, Figure S1).

Bird counts were conducted in 11 distinct land cover types chosen to cover a range of the abundance index classes. A total of 821 bird point counts were conducted in all seasons (except winter) and bird abundance was evaluated in 18 land cover-season units. Between 35 and 56 bird point counts were conducted in each of these 18 land cover-season units and resulted in 506 assessments of specific bird abundance (number of birds contacted in 15 minutes). The mean number of birds of each species was calculated for each land cover-season unit.

The data produced by point-count sessions were used to assess the validity of the expert-based indices of bird species abundance (according to land cover and season). Each bird species * land cover type * season combination was considered as a distinct statistical unit. Bird counts were modeled using abundance index, observer, season, type of habitat (closed *vs.* open), and order to which the species belongs (passerine *vs.* non-passerine) as explanatory variables.

The count data turned out to be zero-inflated and over-dispersed. A zero-inflated negative binomial model was then used to assess the influence of the explanatory variables on the count dependent variable. The explanatory variables considered for the binomial component of the model (representing the probability of the species being present) were ‘abundance index’, ‘season’ and the interaction between ‘abundance index’ and ‘season’. The interaction was included to assess whether the validity of the abundance index was constant over seasons or varied according to the season. The explanatory variables considered for the negative binomial component of the model (representing the number of individuals detected conditionally on the species presence) were ‘abundance index’, ‘season’, ‘order to which the species belongs’, ‘observer’, ‘type of habitat’, and the interaction between ‘abundance index’ and ‘season’. Statistical significance of the explanatory variables was assessed through the p-

values associated model parameters. These p-values are derived from a z-test that assesses the null hypothesis that the parameter value is 0.

Results show that the expert-based indices of bird species abundance are valid to predict the probability of presence of the bird species in the Camargue area. Moreover, the number of detections conditional on species presence depends on the type of habitat and the observer (Tables S5 and S6).

References

Bibby CJ, Burgess ND, Hill DA, Mustoe SH (2000) Bird census techniques. London, Academic press.

Poncon N, Toty C, L'Ambert G, le Goff G, Brengues C, Schaffner F, Fontenille D (2007) Population dynamics of pest mosquitoes and potential malaria and West Nile virus vectors in relation to climatic factors and human activities in the Camargue, France. Med Vet Entomol. 21(4): 350-357.

Schaffner F, Angel G, Geoffroy B, Hervy J-P, Rhaeim A, Brunhes J (2001) The mosquitoes of Europe. Paris, France, IRD Editions and EID Méditerranée.

Sudia WD, Chamberlain RW (1962) Battery-operated light trap, an improved model. Mosquito News. 22: 126-129.

Table S5: Wild bird presence model coefficients

Presence model coefficients					
		Estimate	Std. error	Z value	p value
Intercept		15.85	1.59	9.97	$<10^{-15}$
**Season**	Spring	0	-	-	-
	**Summer**	-5.23	1.42	-3.70	$<10^{-3}$
	Autumn	-2.17	1.63	-1.33	0.18
**Abundance index**	Absent	0	-	-	-
	**Uncommon**	-13.26	1.52	-8.71	$<10^{-15}$
	**Frequent**	-18.63	1.94	-9.62	$<10^{-15}$
	Common	-83.75	49.27	-1.7	0.09
**Interactions**	Autumn*Uncommon	1.86	1.63	1.15	0.25
	**Summer*Uncommon**	8.48	1.55	5.46	$<10^{-7}$
	**Autumn*Frequent**	6.11	2.33	2.62	$<10^{-2}$
	**Summer*Frequent**	8.68	2.08	4.16	$<10^{-4}$
	Autumn*Common	70.27	49.28	1.43	0.15
	**Summer*Common**	51.34	17.97	2.86	$<10^{-2}$

Table S6: Wild bird count model coefficients

Count model coefficients					
		Estimate	Std. error	Z value	p value
Intercept		-1.10	0.15	-7.14	$<10^{-12}$
Observer	Obs1	0	-	-	-
	Obs2	0.26	0.08	3.30	$<10^{-3}$
Type of habitat	Close	0	-	-	-
	Open	0.63	0.10	6.60	$<10^{-10}$
Order	Non-passerine	0	-	-	-
	Passerine	0.14	0.09	1.63	0.10
Season	Spring	0	-	-	-
	Summer	1.43	0.11	13.54	$<10^{-15}$
	Autumn	1.00	0.13	7.78	$<10^{-14}$
Abundance index	Absent	0	-	-	-
	Uncommon	0.85	0.13	6.79	$<10^{-10}$
	Frequent	0.55	0.12	4.50	$<10^{-5}$
	Common	0.86	0.15	5.65	$<10^{-7}$
Probability of species' presence	Log(θ)	-1.89	0.07	-28.78	$<10^{-15}$
