# Supplementary material for: An Integrative Eco-Epidemiological Analysis of West Nile Virus Transmission
Source: Ecohealth. 2017 Jun 5;14(3):474–89. doi: 10.1007/s10393-017-1249-6 (PMC5662683; doi:10.1007/s10393-017-1249-6)
Supplement: Supplementary file 5 — Supplementary material 5 (PDF 26 kb) [file 10393_2017_1249_MOESM5_ESM.pdf]

An Integrative Eco-Epidemiological Analysis of West Nile Virus Transmission

Technical Appendix 5. Spatial analysis procedures to predict areas of WNV transmission

5.1. Definition of potential West Nile Virus (WNV) 'introduction areas' *(I)*

The initial 'introduction areas' of WNV in the study area were defined as sites where the virus could be transmitted for the first time in a given year, originating either from migratory birds (hypothesis I_1) or from overwintering mosquitoes (hypothesis I_2). We mapped the 'introduction sites' through the following process:

1) Hypothesis I_{1a}: WNV is introduced in spring by southern bird migrants. The potential for WNV introduction was estimated for each land cover class according to the sum of the abundance indices of southern bird migrant species present in spring, recoded in four classes (0: null; 1: low; 2: moderate; 3: high) the standard-deviation classification method. The standard-deviation method (or 'mean-standard deviation') is a common method of classification used in GIS for the creation of choropleth maps and adapted for map comparison, based on the mean and standard deviation values of the dataset to be classified: class breaks are defined above and below the mean at intervals of multiples of the standard deviation.
2) Hypothesis I_{1b}: WNV is introduced in late summer by eastern bird migrants. The potential for WNV introduction was estimated for each land cover class according to the sum of the abundance indices of eastern bird migrant species present in summer, recoded in four classes (0: null; 1: low; 2: moderate; 3: high) through the standard-deviation classification method.
3) Hypothesis I_2: Mosquitoes could permit the overwintering of WNV in the Camargue area: either *Cx. modestus* (hypothesis I_{2a}) or *Cx. pipiens* (I_{2b}) or both species (I_{2c})

(Balenghien et al., 2008). The 'introduction areas' under this assumption were defined as the areas where mosquito populations were still present in autumn, before entering diapause, as defined in Technical Appendix 3.1.

5.2 Definition of potential West Nile Virus (WNV) 'amplification areas' (*A*)

Virus amplification occurs when a bird-to-bird transmission cycle takes place, the virus being transmitted by mosquitoes. Thus, 'amplification areas' are located where competent host and vector populations intersect (Figure 1), and the level of amplification was defined according to the abundance of vector and host populations and to the composition of the bird community.

We considered that amplification occurs in summer and mapped the 'amplification areas' performing the following steps:

1) *Index of potential virus amplification by the vectors.* Different mosquito species could be competent for WNV amplification: either *Cx. modestus* (hypothesis A_{1xx}) or *Cx. pipiens* (A_{2xx}) or both species (A_{3xx}) (Balenghien, Vazeille et al., 2008). An index of potential virus amplification by the vector population, ranging from 0 to 3, was attributed to each location of the study area according to the abundance of either one (level of risk equal to 0 when the species is absent or present at very low densities and equal to 1, 2 or 3 when the species is present at low, high or very high densities, respectively) or both mosquito species (Table S7).
2) *Index of potential virus amplification by the hosts.* Birds may have a different reservoir status for WNV amplification. House sparrows (*Passer domesticus*) and black-billed magpies (*Pica pica*) are the only two bird species in which the virus was isolated in the Camargue region (Jourdain et al., 2007); they thus could be considered as the only competent hosts in the area (hypothesis A_{x1x}). On the other hand, every bird species could be involved in the WNV transmission cycle, either with different

competences, according to the host competence index (hypothesis A_{x2x}, Table S1), or all with the same competence (hypothesis A_{x3x}). For each hypothesis, an index of potential virus amplification by the hosts was calculated as the weighted sum of the species' abundance indices (Technical Appendix 3.2), with the following weights: 1 for sparrows and magpies and 0 for all other species (A_{x1x}); the value of the host competence index defined in the bird database (A_{x2x}, Table S1); 1 for every species (A_{x3x}). Then, for each hypothesis (A_{x1x}, A_{x2x}, A_{x3x}) this index of potential host amplification was recoded, ranging from 0 to 3 and reflecting the potential WNV amplification by the hosts (classification using the standard-deviation method).

3) *'Dilution effect' index.* The composition of the bird community may impact the amplification of WNV by creating a 'dilution effect', namely when an increase in the abundance of less competent hosts causes a decrease in the infection prevalence in the vector population (Ostfeld and Keesing, 2000; Keesing et al., 2006). We therefore considered two hypotheses: firstly, the bird community composition does not impact virus amplification (hypothesis A_{xx1}: absence of 'dilution effect'); second, an increase of the number of less-competent host causes a decrease of virus amplification (hypothesis A_{xx2}). In this second case, a 'dilution effect' index (0 to 3) was attributed to each location decreasing when the number of less-competent species (with host competence index equal to 1) increases. Under hypothesis A_{xx1}, the 'dilution effect' index was set to one. For each hypothesis (A_{xx1}, A_{xx2}) the 'dilution effect' index was recoded, ranging from 0 to 3 (classification using the standard-deviation method).
4) The potential for virus amplification was finally defined for each location in the study area as the product of the index of potential virus amplification by the vectors, the index of potential virus amplification by the hosts, and the 'dilution effect' index, recoded between 0 and 3 (classification using the standard-deviation method).

5.3. Definition of possible West Nile Virus (WNV) ‘spillover areas’ (*S*)

Both *Culex modestus* and *Cx. pipiens* are likely to act as bridge vectors between birds and mammals in the study area (32, 44), so we considered three hypotheses of emergence. Either *Cx. modestus* is responsible for transmission of WNV to equine population (S_1) or *Cx. pipiens* (S_2) or both species (S_3). Thus, the ‘spillover areas’ were defined as the areas where each or both species are present in summer (with class of abundance higher than 2).

5.4. Calculation of the West Nile Virus (WNV) circulation index

A WNV circulation index was defined as the risk level for WNV circulation in wild birds, after virus introduction, amplification/ dispersal, for each combination of hypotheses tested.

1) The predicted areas of introduction were intersected with the areas of amplification: a level of risk of introduction-amplification for each location of the study area was obtained by the multiplication of the risk of introduction (*I*) and the risk of amplification (*A*), and recoded to range from 0 to 3 (classification using the standard-deviation method).
2) A distance map to each ecological unit where this level of risk of introduction-amplification was not null was generated using the Euclidean distance function (ESRI Spatial Analyst/Distance tools).
3) J-shaped functions (one for each of the six classes of dispersal distance of bird species as defined in the integrative database: <0.5 km, <1 km, <10 km, <50 km, > 50 km) were applied to these distance maps (ESRI Spatial Analyst/Map Algebra/Raster Calculator tool). These functions vary continuously from 1 to 0, taking the value 1 if the distance to the ecological unit considered is null and 0 if this distance is greater than the dispersal distance. This operation resulted in the generation of the potential

areas of WNV dispersal by birds from each ecological unit where introduction and amplification occurred, according to five dispersal range classes.

4) Finally, the WNV circulation index map was obtained by summing the maps of potential areas of WNV dispersal from each ecological unit, weighted by *i)* the level of risk of introduction-amplification calculated in step 1; *ii)* the number of bird species present in the ecological unit with the corresponding dispersal distance, weighted according to the amplification hypothesis (weight = 1 for sparrows and magpies and 0 for all other species (A_{x1x}); the value of the host competence index defined in the bird database (A_{x2x}); 1 for every species (A_{x3x})) (ESRI Spatial Analyst/Map Algebra/Raster Calculator tool).

Table S7: Level of risk of amplification by the vector population attributed to each location according to the abundance of both mosquito species (absent: never collected; very low densities: few individuals collected; low densities: 10 to 40 individuals collected; high densities: 40 to 100 individuals collected; very high densities: >100 individuals collected).

Culex modestus / ***Culex pipiens***	**Absent**	**Very low densities**	**Low densities**	**High densities**	**Very high densities**
**Absent**	0	0	0	1	2
**Very low densities**	0	1	1	2	3
**Low densities**	0	1	2	3	3
**High densities**	1	2	3	3	3
**Very high densities**	2	3	3	3	3

References

Balenghien T, Vazeille M, Grandadam M, Schaffner F, Zeller H, Reiter P, Sabatier P, Fouque F, Bicout DJ (2008) Vector competence of some French Culex and Aedes mosquitoes for West Nile virus. Vector Borne and Zoonotic Diseases 8(5): 589-595.

Jourdain E, Schuffenecker I, Korimbocus J, Reynard S, Murri S, Kayser Y, Gauthier-Clerc M, Sabatier P, Zeller HG (2007) West Nile virus in wild resident birds, Southern France, 2004. Vector Borne Zoonotic Dis. 7(3): 448-452.

Keesing F, Holt RD, Ostfeld RS (2006) Effects of species diversity on disease risk. Ecology Letters. 9(4): 485-498.

Ostfeld R, Keesing F (2000) The function of biodiversity in the ecology of vector-borne zoonotic diseases. Canadian Journal of Zoology-Revue Canadienne De Zoologie. 78(12): 2061-2078.
