# Supplementary material for: An Integrative Eco-Epidemiological Analysis of West Nile Virus Transmission
Source: Ecohealth. 2017 Jun 5;14(3):474–89. doi: 10.1007/s10393-017-1249-6 (PMC5662683; doi:10.1007/s10393-017-1249-6)
Supplement: Supplementary file 6 — Supplementary material 6 (PDF 40 kb) [file 10393_2017_1249_MOESM6_ESM.pdf]

An Integrative Eco-Epidemiological Analysis of West Nile Virus Transmission

Technical Appendix 6. Results.

Table S8: List of all regression models evaluating the variation in seroprevalence in magpies in relation to West Nile Virus circulation index, resulting from the different scenarios of introduction and amplification/dispersal, Camargue area, Southern France, and ranked according to their normalized Akaike weights (w_{AIC})

Scenario	Introduction[1]	Amplification			AIC	w_{AIC}
		Vector[2]	Host[3]	'Dilution effect'[4]		
I1bA321	1b	3	2	1	171.319	0.296
I1aA121	1a	1	2	1	173.030	0.126
I1bA121	1b	1	2	1	173.645	0.092
I1aA321	1a	3	2	1	173.931	0.080
I2cA122	2c	1	2	2	173.967	0.079
I2bA321	2b	3	2	1	174.729	0.054
I2cA121	2c	1	2	1	174.930	0.049
I2bA222	2b	2	2	2	175.590	0.035
I2cA321	2c	3	2	1	175.683	0.033
I2cA322	2c	3	2	2	175.915	0.030
I2cA222	2c	2	2	2	176.183	0.026
I1aA221	1a	2	2	1	176.220	0.026
I2bA221	2b	2	2	1	176.847	0.019
I2bA121	2b	1	2	1	176.852	0.019
I2cA221	2c	2	2	1	177.300	0.015
I1aA222	1a	2	2	2	177.385	0.014
I1bA322	1b	3	2	2	179.339	0.005
I2bA122	2b	1	2	2	181.319	0.002
I1bA122	1b	1	2	2	183.783	0.001
I1aA122	1a	1	2	2	184.749	0.000
I1aA322	1a	3	2	2	185.099	0.000
I1bA131	1b	1	3	1	186.233	0.000
I1bA331	1b	3	3	1	186.233	0.000
I1aA131	1a	1	3	1	186.353	0.000

[1] 1a: virus introduction by migratory birds: Southern spring migrants; 1b: virus introduction by migratory birds, Eastern summer migrants; 2a: Virus overwintering in vectors: *Culex modestus*; 2b: Virus overwintering in vectors: *Cx. pipiens*; 2c : Virus overwintering in vectors: *Cx. modestus* and *Cx. pipiens*

[2] 1: *Cx. modestus*; 2: *Cx. pipiens*; 3: *Cx. modestus* and *Cx. pipiens*

[3] 1: House sparrow and black-billed magpies only; 2: All species, heterogeneous competences; 3 : All species, homogeneous competences

[4] 1: No 'dilution' effects; 2 : Risk decreases with the number of non-competent species ('dilution effect')

Scenario	Introduction[5]	Amplification			AIC	w_{AIC}
		Vector[6]	Host[7]	'Dilution effect'[8]		
I2aA221	2a	2	2	1	189.053	0.000
I2aA222	2a	2	2	2	189.053	0.000
I2aA231	2a	2	3	1	193.361	0.000
I2aA321	2a	3	2	1	193.396	0.000
I2aA322	2a	3	2	2	193.396	0.000
I2aA121	2a	1	2	1	193.432	0.000
I2aA122	2a	1	2	2	193.432	0.000
I2aA131	2a	1	3	1	193.432	0.000
I2aA331	2a	3	3	1	193.432	0.000
I2bA131	2b	1	3	1	193.432	0.000
I2cA131	2c	1	3	1	193.432	0.000
I1bA221	1b	2	2	1	194.305	0.000
I1bA222	1b	2	2	2	195.082	0.000
I1aA111	1a	1	1	1	196.903	0.000
I1aA112	1a	1	1	2	196.903	0.000
I2bA322	2b	3	2	2	197.093	0.000
I1bA212	1b	2	1	2	203.955	0.000
I1aA212	1a	2	1	2	204.008	0.000
I1aA312	1a	3	1	2	204.008	0.000
I2bA212	2b	2	1	2	204.008	0.000
I2bA312	2b	3	1	2	204.008	0.000
I2cA212	2c	2	1	2	204.008	0.000
I2cA312	2c	3	1	2	204.008	0.000
I1bA112	1b	1	1	2	204.247	0.000
I1bA312	1b	3	1	2	204.247	0.000
I2aA112	2a	1	1	2	204.247	0.000
I2aA212	2a	2	1	2	204.247	0.000
I2aA312	2a	3	1	2	204.247	0.000
I2bA112	2b	1	1	2	204.247	0.000
I2cA112	2c	1	1	2	204.247	0.000
I1bA231	1b	2	3	1	206.641	0.000
I1bA211	1b	2	1	1	208.644	0.000
I1aA211	1a	2	1	1	208.673	0.000
I2bA211	2b	2	1	1	208.673	0.000
I2cA211	2c	2	1	1	208.673	0.000
I1aA331	1a	3	3	1	209.247	0.000

[5] 1a: virus introduction by migratory birds: Southern spring migrants; 1b: virus introduction by migratory birds, Eastern summer migrants; 2a: Virus overwintering in vectors: *Culex modestus*; 2b: Virus overwintering in vectors: *Cx. pipiens*; 2c : Virus overwintering in vectors: *Cx. modestus* and *Cx. pipiens*

[6] 1: *Cx. modestus*; 2: *Cx. pipiens*; 3: *Cx. modestus* and *Cx. pipiens*

[7] 1: House sparrow and black-billed magpies only; 2: All species, heterogeneous competences; 3 : All species, homogeneous competences

[8] 1: No 'dilution' effects; 2 : Risk decreases with the number of non-competent species ('dilution effect')

Scenario	Introduction[9]	Amplification			AIC	w_{AIC}
		Vector[10]	Host[11]	'Dilution effect'[12]		
I2aA211	2a	2	1	1	209.709	0.000
I1aA231	1a	2	3	1	209.864	0.000
I2bA231	2b	2	3	1	209.864	0.000
I2cA231	2c	2	3	1	209.864	0.000
I2bA331	2b	3	3	1	209.878	0.000
I2cA331	2c	3	3	1	209.878	0.000
I1aA311	1a	3	1	1	209.932	0.000
I2bA311	2b	3	1	1	209.932	0.000
I2cA311	2c	3	1	1	209.932	0.000
I1bA111	1b	1	1	1	210.006	0.000
I1bA311	1b	3	1	1	210.006	0.000
I2aA111	2a	1	1	1	210.006	0.000
I2aA311	2a	3	1	1	210.006	0.000
I2bA111	2b	1	1	1	210.006	0.000
I2cA111	2c	1	1	1	210.006	0.000

[9] 1a: virus introduction by migratory birds: Southern spring migrants; 1b: virus introduction by migratory birds, Eastern summer migrants; 2a: Virus overwintering in vectors: *Culex modestus*; 2b: Virus overwintering in vectors: *Cx. pipiens*; 2c : Virus overwintering in vectors: *Cx. modestus* and *Cx. pipiens*

[10] 1: *Cx. modestus*; 2: *Cx. pipiens*; 3: *Cx. modestus* and *Cx. pipiens*

[11] 1: House sparrow and black-billed magpies only; 2: All species, heterogeneous competences; 3 : All species, homogeneous competences

[12] 1: No 'dilution' effects; 2 : Risk decreases with the number of non-competent species ('dilution effect')

Table S9: List of all regression models evaluating the variation in seroprevalence in equines in relation to West Nile Virus spillover index, resulting from the different scenarios of introduction, amplification/dispersal and spillover, Camargue area, Southern France, and ranked according to their normalized Akaike weights (w_{AIC})

Scenario	Introduction[13]	Amplification			Spillover[†]	AIC	w_{AIC}
		Vector[14]	Host[15]	'Dilution effect'[16]			
I1aA121S3	1a	1	2	1	3	725.539	0.568
I1bA131S3	1b	1	3	1	3	728.330	0.141
I1bA331S3	1b	3	3	1	3	728.330	0.141
I1aA131S3	1a	1	3	1	3	729.403	0.082
I2aA121S3	2a	1	2	1	3	732.239	0.020
I2aA122S3	2a	1	2	2	3	732.239	0.020
I1aA121S2	1a	1	2	1	2	736.208	0.003
I1aA321S3	1a	3	2	1	3	736.428	0.002
I1aA121S1	1a	1	2	1	1	737.328	0.002
I1bA131S1	1b	1	3	1	1	737.423	0.001
I1bA331S1	1b	3	3	1	1	737.423	0.001
I1aA122S1	1a	1	2	2	1	737.743	0.001
I1bA122S1	1b	1	2	2	1	738.017	0.001
I2aA321S3	2a	3	2	1	3	738.555	0.001
I2aA322S3	2a	3	2	2	3	738.555	0.001
I1aA322S1	1a	3	2	2	1	738.585	0.001
I1bA321S3	1b	3	2	1	3	738.594	0.001
I2aA131S3	2a	1	3	1	3	738.684	0.001
I2aA231S3	2a	2	3	1	3	738.684	0.001
I2aA331S3	2a	3	3	1	3	738.684	0.001
I2bA131S3	2b	1	3	1	3	738.684	0.001
I2cA131S3	2c	1	3	1	3	738.684	0.001
I2cA121S3	2c	1	2	1	3	738.801	0.001
I1aA321S1	1a	3	2	1	1	738.928	0.001
I1aA131S1	1a	1	3	1	1	739.007	0.001
I1bA322S1	1b	3	2	2	1	739.096	0.001
I2aA121S2	2a	1	2	1	2	739.099	0.001
I2aA122S2	2a	1	2	2	2	739.099	0.001
I2bA121S3	2b	1	2	1	3	739.642	0.000
I1bA321S1	1b	3	2	1	1	739.645	0.000

[13] 1a: virus introduction by migratory birds: Southern spring migrants; 1b: virus introduction by migratory birds, Eastern summer migrants; 2a: Virus overwintering in vectors: *Culex modestus*; 2b: Virus overwintering in vectors: *Cx. pipiens*; 2c : Virus overwintering in vectors: *Cx. modestus* and *Cx. pipiens*

[14] 1: *Cx. modestus*; 2: *Cx. pipiens*; 3: *Cx. modestus* and *Cx. pipiens*

[15] 1: House sparrow and black-billed magpies only; 2: All species, heterogeneous competences; 3 : All species, homogeneous competences

[16] 1: No 'dilution' effects; 2 : Risk decreases with the number of non-competent species ('dilution effect')

Scenario	Introduction[17]	Amplification			Spillover[†]	AIC	w_{AIC}
		Vector[18]	Host[19]	'Dilution effect'[20]			
I2cA121S1	2c	1	2	1	1	741.188	0.000
I1aA222S3	1a	2	2	2	3	741.442	0.000
I2aA121S1	2a	1	2	1	1	741.517	0.000
I2aA122S1	2a	1	2	2	1	741.517	0.000
I2bA121S1	2b	1	2	1	1	741.766	0.000
I1bA122S3	1b	1	2	2	3	741.853	0.000
I1aA222S1	1a	2	2	2	1	741.873	0.000
I1aA122S3	1a	1	2	2	3	741.929	0.000
I1aA221S3	1a	2	2	1	3	742.116	0.000
I1aA221S1	1a	2	2	1	1	742.433	0.000
I1aA131S2	1a	1	3	1	2	742.646	0.000
I2cA122S1	2c	1	2	2	1	742.662	0.000
I1bA131S2	1b	1	3	1	2	742.763	0.000
I1bA331S2	1b	3	3	1	2	742.763	0.000
I2aA321S1	2a	3	2	1	1	742.903	0.000
I2aA322S1	2a	3	2	2	1	742.903	0.000
I2aA131S1	2a	1	3	1	1	742.949	0.000
I2aA231S1	2a	2	3	1	1	742.949	0.000
I2aA331S1	2a	3	3	1	1	742.949	0.000
I2bA131S1	2b	1	3	1	1	742.949	0.000
I2cA131S1	2c	1	3	1	1	742.949	0.000
I1aA322S3	1a	3	2	2	3	743.357	0.000
I1aA222S2	1a	2	2	2	2	744.574	0.000
I2cA121S2	2c	1	2	1	2	744.662	0.000
I2aA321S2	2a	3	2	1	2	744.701	0.000
I2aA322S2	2a	3	2	2	2	744.701	0.000
I2aA131S2	2a	1	3	1	2	744.808	0.000
I2aA231S2	2a	2	3	1	2	744.808	0.000
I2aA331S2	2a	3	3	1	2	744.808	0.000
I2bA131S2	2b	1	3	1	2	744.808	0.000
I2cA131S2	2c	1	3	1	2	744.808	0.000
I2cA122S3	2c	1	2	2	3	745.224	0.000
I1aA221S2	1a	2	2	1	2	745.327	0.000
I1bA322S3	1b	3	2	2	3	745.491	0.000
I2bA121S2	2b	1	2	1	2	745.702	0.000
I1bA121S1	1b	1	2	1	1	746.759	0.000
I2cA221S1	2c	2	2	1	1	747.296	0.000

[17] 1a: virus introduction by migratory birds: Southern spring migrants; 1b: virus introduction by migratory birds, Eastern summer migrants; 2a: Virus overwintering in vectors: *Culex modestus*; 2b: Virus overwintering in vectors: *Cx. pipiens*; 2c : Virus overwintering in vectors: *Cx. modestus* and *Cx. pipiens*
[18] 1: *Cx. modestus*; 2: *Cx. pipiens*; 3: *Cx. modestus* and *Cx. pipiens*
[19] 1: House sparrow and black-billed magpies only; 2: All species, heterogeneous competences; 3 : All species, homogeneous competences
[20] 1: No 'dilution' effects; 2 : Risk decreases with the number of non-competent species ('dilution effect')

Scenario	Introduction[21]	Amplification			Spillover[†]	AIC	w_{AIC}
		Vector[22]	Host[23]	'Dilution effect'[24]			
I1bA321S2	1b	3	2	1	2	747.530	0.000
I2bA221S1	2b	2	2	1	1	748.837	0.000
I2cA222S1	2c	2	2	2	1	749.363	0.000
I2cA122S2	2c	1	2	2	2	749.399	0.000
I2cA221S3	2c	2	2	1	3	750.118	0.000
I2aA221S1	2a	2	2	1	1	750.577	0.000
I2aA222S1	2a	2	2	2	1	750.577	0.000
I2bA222S1	2b	2	2	2	1	751.629	0.000
I1aA321S2	1a	3	2	1	2	751.802	0.000
I2cA221S2	2c	2	2	1	2	752.050	0.000
I2bA221S3	2b	2	2	1	3	752.308	0.000
I2cA222S3	2c	2	2	2	3	752.740	0.000
I2bA221S2	2b	2	2	1	2	753.852	0.000
I2cA222S2	2c	2	2	2	2	754.058	0.000
I2aA221S3	2a	2	2	1	3	754.450	0.000
I2aA222S3	2a	2	2	2	3	754.450	0.000
I1bA122S2	1b	1	2	2	2	755.294	0.000
I1aA122S2	1a	1	2	2	2	755.379	0.000
I2bA222S3	2b	2	2	2	3	755.947	0.000
I1aA322S2	1a	3	2	2	2	756.004	0.000
I1bA322S2	1b	3	2	2	2	756.015	0.000
I1bA121S3	1b	1	2	1	3	756.538	0.000
I2aA221S2	2a	2	2	1	2	756.650	0.000
I2aA222S2	2a	2	2	2	2	756.650	0.000
I2bA222S2	2b	2	2	2	2	756.970	0.000
I1bA121S2	1b	1	2	1	2	759.465	0.000

[21] 1a: virus introduction by migratory birds: Southern spring migrants; 1b: virus introduction by migratory birds, Eastern summer migrants; 2a: Virus overwintering in vectors: *Culex modestus*; 2b: Virus overwintering in vectors: *Cx. pipiens*; 2c : Virus overwintering in vectors: *Cx. modestus* and *Cx. pipiens*
[22] 1: *Cx. modestus*; 2: *Cx. pipiens*; 3: *Cx. modestus* and *Cx. pipiens*
[23] 1: House sparrow and black-billed magpies only; 2: All species, heterogeneous competences; 3 : All species, homogeneous competences
[24] 1: No 'dilution' effects; 2 : Risk decreases with the number of non-competent species ('dilution effect')
